# Warm (for Winter): Inferring Comparison Classes in Communication

**DOI:** 10.1111/cogs.13095

**Published:** 2022-03-16

**Authors:** Michael Henry Tessler, Noah D. Goodman

**Affiliations:** ^1^ Department of Brain and Cognitive Sciences Massachusetts Institute of Technology; ^2^ Department of Psychology Stanford University; ^3^ Department of Computer Science Stanford University

**Keywords:** Context | Adjectives, Bayesian cognitive model, Bayesian data analysis, Comparison class, Pragmatics, Reference class, Rational Speech Act

## Abstract

The meanings of natural language utterances depend heavily on context. Yet, what counts as context is often only implicit in conversation. The utterance *it's warm outside* signals that the temperature outside is relatively high, but the temperature could be high relative to a number of different *comparison classes*: other days of the year, other weeks, other seasons, etc. Theories of context sensitivity in language agree that the comparison class is a crucial variable for understanding meaning, but little is known about how a listener decides upon the comparison class. Using the case study of gradable adjectives (e.g., *warm*), we extend a Bayesian model of pragmatic inference to reason flexibly about the comparison class and test its qualitative predictions in a large‐scale free‐production experiment. We find that human listeners infer the comparison class by reasoning about the kinds of observations that would be remarkable enough for a speaker to mention, given the speaker and listener's shared knowledge of the world. Further, we quantitatively synthesize the model and data using Bayesian data analysis, which reveals that usage frequency and a preference for basic‐level categories are two main factors in comparison class inference. This work presents new data and reveals the mechanisms by which human listeners recover the relevant aspects of context when understanding language.

## 1 Introduction

A $75^{\circ }$F ($24^{\circ }$C) day is warm. A $50^{\circ }$F ($10^{\circ }$C) day is not. That is, unless it is Winter; $50^{\circ }$F ($10^{\circ }$C) could be warm for Winter. Whether or not something is *warm* depends upon what *reference class* you consider: a warm Winter day is different than a typical warm day of the year. Deciding upon a *reference class* is an open‐ended problem, articulated most explicitly in the philosophy of probability when one tries to compute a probability for a singular future event (Hájek, 2007; Reichenbach, 1949): Any singular event or entity belongs to multiple categories, giving rise to multiple possible reference classes. For example, to calculate the probability that Joe Biden will be reelected President of the United States in 2024, one would ideally construct a set of similar past events and compute the frequency of successful outcomes (e.g., reelections) relative to the total number of outcomes (number of elections); but, what “similar” past events should be considered? There is the set of all elections involving older white males at the state and federal level in Western democracies between the years 1890–2020, the set of elections of U.S. Presidents born in Pennsylvania, the set of elections of heads‐of‐state who have governed during global pandemics, among countless other possibilities. Reference classes are deployed in contexts as diverse as providing a price valuation for a house (e.g., by appealing to the prices of “similar” houses) to informing the severity of sentences for defendants convicted in the legal system (e.g., by appealing to sentences of “similar” convictions; Cheng, 2009; Colyvan, Regan, & Ferson, 2001). Even in ordinary language, the reference class problem is present (e.g., what do you mean by *warm*?), and yet human speakers and listeners seem to fill in this critical aspect of context with hardly a notice.

In natural language, the problem of reference or comparison classes^1^ is front and center when interpreting *relative* statements (e.g., *warm relative to what?*). The problem thus affects a wide swath of linguistic expressions, including adjectives (*warm*, *tall*; Bartsch & Vennemann, 1972; Cresswell, 1976; Kennedy & McNally, 2005; Klein, 1980), quantifiers (e.g., *many*, *a lot*; Schöller & Franke, 2017), and, in a nonobvious way, category generalizations such as generic or habitual statements (e.g., *Robins lay eggs*, *John smokes*; Tessler & Goodman, 2019). Formally, the comparison class is a free (underspecified) variable that linguistic theories assume is filled in by context (Bale, 2008, 2011; Kennedy & McNally, 2005; Solt, 2009). Psychological and/or communicative mechanisms presumably are responsible for that “filling in” process, but how those mechanisms operate to determine a comparison class remains obscure. Comparison classes thus also serve as a case study of representations at the interface of psychological and linguistic theory.

Empirically, the question of how human listeners decide upon the comparison class has received little systematic attention. Empirical work with adults and children has primarily interrogated how judgments and interpretations of relative adjectives (e.g., *dark*, *tall*) depend upon the statistical details of a predetermined comparison class (Barner & Snedeker, 2008; Qing & Franke, 2014b; Schmidt, Goodman, Barner, & Tenenbaum, 2009; Solt & Gotzner, 2012). Indirect evidence that the comparison class is flexibly inferred comes from studies with young children, which find that a directly modified noun phrase (e.g., *tall pimwit*, where *pimwit* is a novel category label) can be used to constrain the kinds of objects that go into the comparison class: What counts as a *tall pimwit* depends on the distribution of heights of *pimwits* and not the heights of other categories like *daxes* (i.e., *daxes* are not included in the comparison class; Barner & Snedeker, 2008). Additionally, strong linguistic and perceptual cues can provide a signal to children as young as two‐and‐a‐half that the comparison class can change (e.g., an objectively small mitten can be *big*, if there are many tiny mittens on the table; Ebeling & Gelman, 1988, 1994). Still, a systematic investigation into how listeners flexibly adjust the comparison class is outstanding.

In what follows, we examine the problem of inferring comparison classes in communication using gradable adjectives like *warm* or *tall* as our case study. We describe how comparison classes can be incorporated into computational models of pragmatic communication. The model predicts an intuitive interaction between a listener's world knowledge (e.g., winter days are generally cold) and the valence (or, polarity) of the adjective heard (e.g., *warm* vs. *cold*), which contrasts with the predictions of a literal Bayesian reasoner model. The pragmatic model also makes fine‐grained quantitative predictions of comparison class inferences as a function of distributional knowledge about the property in the category (e.g., plausible temperatures of Winter days) and prior beliefs about the comparison class. We test these predictions in a preregistered, large‐scale free‐production experiment in which participants freely generate comparison classes when interpreting relative adjectives in context. We further investigate the structure of the comparison class prior and show that it credibly contains elements that encode a preference for basic‐level categories (Rosch & Mervis, 1975) and category labels that are more common in everyday speech (i.e., usage frequency).

## 2 Computational model

Here we articulate the computations that underly comparison class inference in communication and how that inference interfaces with standard linguistic theories of adjective meaning. When interpreting an adjectival utterance u which lacks an explicit comparison class c (e.g., *he's tall* [relative to other c]), a listener is faced with the joint inference problem of determining the comparison class c and the property value (or, degree) x described by the adjective (e.g., the height of the referent). A listener $L_{1}$ can use their knowledge of the referent k (e.g., the category membership of the referent, such as that the referent is a basketball player) to guide both their expectations about the property value P(x∣k) (e.g., the plausible height of a basketball player) as well as the hypothesis space of comparison classes P(c∣k) (described in more detail below). A Bayesian listener would infer the likely property value (e.g., height) of the referent x and the likely comparison class c by combining these prior expectations with the likelihood that a speaker $S_{1}\left(u|x,c\right)$ would bother to say that the adjective applies to the referent (i.e., would the speaker bother to say that the referent is tall).

(1) $$\begin{array}{cc} L_{1}\left(x,c|u,k\right) & \propto S_{1}\left(u|x,c\right)\cdot P\left(x|k\right)\cdot P\left(c|k\right). \end{array}$$

**Fig 1:**
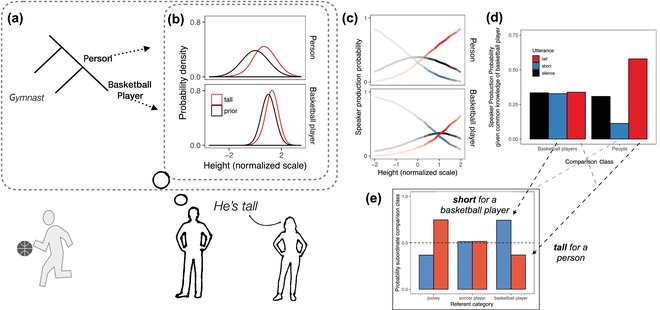
Model overview for the example of a listener hearing a speaker describe a basketball player as *tall*. (A) A hypothesis space of comparison classes is constructed over a taxonomic hierarchy. (B) A comparison class is realized as a probability distribution over the relevant degree (e.g., height; shown in black). Context‐specific probabilistic interpretations of the gradable adjective *tall*—given by $L_{0}\left(x|u,c\right)$—are shown in red for the different comparison classes (facets). (C) Listener $L_{1}$ imagines what a speaker $S_{1}$ would say given different possible heights of the referent (*x*‐axis) assuming different comparison classes (facets); since the listener knows the referent is a basketball player, though, heights towards the upper range of the scale are a priori more likely (opacity). (D) Marginalizing over the a priori plausible heights of the referent, the speaker has a preference to say *tall* over *short* if the comparison class is *other* *people*. (E) The pragmatic listener inverts this speaker model to infer that *people* is the more likely comparison class given that the speaker said *tall*. If the referent is described as *short*, however, the listener infers the speaker meant *short for a basketball player*. Schematic prior distribution of heights for people is a unit normal distribution N(0,1), and the heights of basketball players is a right‐shifted normal with smaller variance N(0.5,0.5).

Equation 1 describes a pragmatic listener $L_{1}$ updating their beliefs about the degree x and the comparison class c by assuming that the speaker $S_{1}$ intentionally produced an adjectival utterance u in order to accurately communicate about the degree (e.g., height). The prior distribution of comparison classes P(c∣k) is assumed to be in common ground (i.e., known to both speaker and listener) and constrained by prior knowledge about categories and the situation (e.g.,  the situation that the referent is a basketball player; the category knowledge that a basketball player is a person; etc.). Comparison classes can be constructed in various ways, including sets of objects in the perceptual environment (e.g., *big relative to the things around it*) or from the hypothetical functions of an object (e.g., *this shirt is big for the doll;* Ebeling & Gelman, 1994); for simplicity, we restrict our analysis to a hypothesis space of comparison classes constructed out of a taxonomic hierarchy (i.e., *conceptual comparison classes*), with the lowest level of hierarchy being the subordinate category to which the referent belongs (e.g., a basketball player; Fig. 1A). The kind of property values (or, degrees) under discussion (e.g., heights) is given by the semantics of the adjectives (e.g., *tall* → height), and the hypothesis space of degrees is informed by the category membership of the referent k (e.g., plausible heights of basketball players).^2^

Following work in the Rational Speech Act (RSA) modeling framework (Frank & Goodman, 2012; Goodman & Frank, 2016; Scontras, Tessler, & Franke, 2018, 2021), the speaker $S_{1}$ in this model is a soft‐max rational agent (with a degree of rationality α) who produces utterances u in order to convey information about the degree x to a listener $L_{0}$.

(3) $$S_{1}\left(u|x,c\right)\propto \exp\left(\alpha \cdot \left(\ln L_{0}\left(x|u,c\right)-\mathrm{cost}\left(u\right)\right)\right).$$

The listener $L_{0}\left(x|u,c\right)$ in the mind of the speaker $S_{1}\left(u|x,c\right)$ is assumed to know the comparison class and is simply a mapping (described below) from prior beliefs about the degree x (e.g., height) to posterior beliefs about x given an adjectival utterance that contains an explicit comparison class (e.g., *tall for a basketball player*). The speaker, as a rational actor, balances the information conveyed by the utterance, $\ln L_{0}\left(x|u,c\right)$, with the cost of producing the utterance u when deciding what to say.^3^ We assume the speaker has three utterances she can say: {*tall*, *short*, silence}, where silence is a semantically vacuous utterance (i.e., a null action).^4^

The listener who updates their beliefs about the degree (e.g., temperature) given a vague adjectival utterance and a fixed comparison class, $L_{0}\left(x|u,c\right)$, is model of context‐sensitive adjective interpretation (Lassiter & Goodman, 2013, 2017; Qing & Franke, 2014a). We use a model of a literal listener which, following standard treatment in formal semantics (e.g., Kennedy, 2007), takes the literal meaning of a gradable adjective to be simply a threshold function on the degree (e.g., [[tall]]=x>θ) and is used to update the listener's prior beliefs about the degree in the comparison class P(x∣c) to produce an interpretation of the adjective, contextualized by the comparison class (Fig. 1B).^5^

(4) $$\begin{array}{cc} L_{0}\left(x,\theta |u,c\right) & \propto \delta_{\left[\;[u]\;](x,\theta \right)}\cdot P\left(x|c\right)\cdot P\left(\theta \right) \end{array}$$

(5) $$\begin{array}{cc} L_{0}\left(x|u,c\right) & =\int_{\theta }L_{0}\left(x,\theta |u,c\right)\;d\theta . \end{array}$$

Equation 4 is a model of literal listener who updates their prior beliefs about the degree given a comparison class P(x∣c) via a threshold function, represented by the Kronecker delta function $\delta_{\left[\;[u]\;\right]}\left(x,\theta \right)$ that returns 1 when the utterance is true (i.e., when x>θ) and 0 otherwise. Lassiter and Goodman (2013, 2017) showed how the context sensitivity of gradable adjectives can be modeled as uncertainty about the threshold P(θ) (where θ comes from a uniform prior distribution over the support of the degree prior), which we adopt here in our literal listener model.^6^ Finally, we assume the communicative goal of using an adjective like *tall* is to convey information about the height of the referent x; thus, the speaker model $S_{1}$ (Eq. 3) chooses utterances to convey the height x to the literal listener $L_{0}$, which we calculate by marginalizing out the threshold variable θ (Eq. 5).

For convenience, we consider an idealized case where there are only two comparison classes: a relatively subordinate‐level category ($c_{sub}$) or a relatively superordinate‐level category ($c_{super}$) (e.g., tall relative to *basketball players* or relative to *people*): $c\in \{c_{sub},c_{super}\}$. The choice of hierarchically structured categories is convenient because subordinate category membership entails the superordinate category membership, but all that we assume in our modeling is that both categories have some nonzero prior probability of being the comparison class. Fig. 1C shows the speaker production probabilities of the three possible utterances (*tall*, *short*, silence) for each value along the degree scale (e.g., each height) for each of the two different comparison classes (facets). If the comparison class is *basketball players*, the speaker will be reluctant to produce *tall* unless the height of the referent is substantially greater than that of the average basketball player (red line shifted to the right with respect to the *person* comparison class). The listener uses Bayes' rule to “invert” this generative model of the utterance (i.e., the speaker model), inferring the implicit comparison class. In doing so, the listener deploys their prior knowledge about the height of the referent: The listener knows the referent is a basketball player, and thus their height is distributed according to the distribution of heights for basketball players (Fig. 1B, bottom, black; this distribution is superimposed as an opacity on Fig. 1C). Averaging over the plausible heights of a basketball player, the listener reasons that a speaker who says *tall* would be more likely to do so assuming the comparison class of *other* *people* (Fig. 1D). Thus, the listener who hears the basketball player described as *tall* tends to think the speaker meant *tall for a person*, whereas the listener who hears a jockey described as *tall* tends to think the speaker meant *tall for a jockey* (Fig. 1E). Overall, the model predicts an intuitive interaction, wherein a listener infers a more superordinate comparison class when the polarity of adjective (e.g., *tall* vs. *short*) is consistent with the listener's background knowledge about the category (e.g., the heights of basketball players vs. jockeys, respectively) than when it is inconsistent.

In our model, a pragmatic listener reasons about what comparison class a speaker would be more likely to assume. One might wonder whether such social reasoning is necessary, or whether these inferences could result from some simpler, non‐social Bayesian reasoning. We examine this question by reformulating the comparison class inference formalized by the pragmatic listener (Eq. 1) in terms of a literal listener model that updates its beliefs about the world and the comparison class only via the literal meaning of the utterance and not via pragmatic reasoning (a la Eq. 4).

(6) $$\begin{array}{c} L_{0}\left(x,\theta ,c|u,k\right)\propto \delta_{\left[\;[u]\;](x,\theta \right)}\cdot P\left(x|c\right)\cdot P\left(\theta \right)\cdot P\left(c|k\right). \end{array}$$

**Fig 2:**
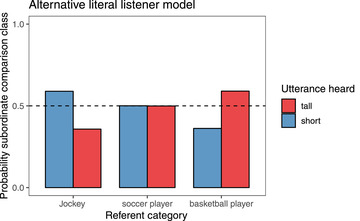
Predictions of the alternative model of a literal listener that does not represent a speaker's representation of the context as separate from their own. This listener effectively answers the question of what is more likely: a basketball player who is tall or a person who is tall?

Similar to the pragmatic listener model (Eq. 1), this listener knows the category membership of the referent k and can use this knowledge to constrain the hypothesis space of comparison classes via P(c∣k) (e.g., with the knowledge that the referent is a basketball player, the listener considers comparison classes that are the same as or superordinate to the class of basketball players). Unlike the pragmatic listener model (Eq. 1), however, the literal listener version of the model does not simultaneously hold different representations of the referent in mind: The pragmatic listener has both their private representation of the referent—which informs the prior distribution of the degree P(x∣k)—and imagines a speaker who acts by assuming some comparison class is in common ground—$S_{1}\left(u|c\right)$—where c and k may or may not refer to the same class (e.g., the listener may know the referent is a basketball player—k=basketballplayers—but believes the speaker was assuming a *person* comparison class—c=people). The literal listener version of the model has no way of separating these representations. This alternative literal listener model also predicts an interaction between background knowledge and the adjective polarity, but the directionality of these effects is exactly the opposite from the predictions of the pragmatic model (Fig. 2).

## 3 Experiment

We test the model's main prediction that the comparison class can be flexibly adjusted based on world knowledge and pragmatic principles in a large‐scale, web‐based free‐production experiment. In the experiment, participants rephrase a speaker's statement, which involves a scalar adjective, in a way that makes the comparison class explicit. (A smaller‐scale, forced‐choice version of this task was reported in Tessler, Lopez‐Brau, and Goodman (2017).) To norm the world knowledge needed in our quantitative modeling, we also measure truth judgments for statements involving scalar adjectives and an explicit comparison class (described in more below and described fully in the Supporting Information [SI]). Sample size, exclusion criteria, regression analysis, and cognitive model analysis were preregistered for the comparison class inference task: osf.io/xuc96. All data reported, analysis scripts, code for cognitive models, and web experiments can be found at github.com/mhtess/comparison‐class‐paper.

### 3.1 Methods

#### 3.1.1 Participants

We recruited 837 participants from Amazon's Mechanical Turk. This number was arrived at with the goal of estimating the probability of a subordinate versus superordinate comparison class (in a 2AFC setting) with confidence intervals no larger than 0.20 for each unique item in the dataset, which conservatively produced an estimate of roughly 50 responses per item.

Participants were restricted to those with U.S. IP addresses with at least a 95% work approval rating. In addition, participants were required to pass a simple language comprehension test that we designed in order to weed out bots and other bad‐faith participants (see the SI). Participants who failed this check were required to exit the experiment, and so we do not have an estimate for how many participants failed this check.

#### 3.1.2 Materials

The experiment involved the interpretation of gradable adjectives that describe physical dimensions (15 adjective pairs in total, e.g., *tall*/*short*; *warm*/*cold*; see Table 1 for full list). The categories (nouns) used in the experiment were a modified subset of a set of adjective–noun pairs generated by a separate group of participants (n=50) in a task designed to elicit stimuli for this experiment (see the SI for details). From this set of participant‐generated stimuli, we curated 90 item sets, each composed of three categories at the same level of abstraction (e.g., they were all subordinate categories of the same basic‐level category) that were sensible in the contexts we used in the comparison class inference experiment. Two examples from each adjective pair are shown in Table 1.

**Table 1:** Thirty of the ninety sets of adjectives and categories used in the comparison class inference experiment. Categories were curated from a set of empirically elicited noun phrases from a stimulus generation task (see the SI)

Adjectives (Scale)	Example Subordinate Classes (Superordinate Class)
*big*, *small* (size)	*great dane*, *poodle*, *chihuahua* (dogs)
	*elephant*, *monkey*, *mouse* (animals)
*tall*, *short* (height)	*basketball player*, *golfer*, *jockey* (people)
	*redwood*, *alpine*, *bonsai* (trees)
*expensive*, *cheap* (price)	*boots*, *sneakers*, *sandals* (footwear)
	*steakhouse*, *buffet*, *diner* (restaurants)
*warm*, *cold* (temperature)	*summer*, *fall*, *winter* (seasons)
	*soup*, *salad*, *ice cream* (food)
*hot*, *cold* (temperature)	*coffee*, *juice*, *milkshake* (drinks)
	*sauna*, *shopping mall*, *ice rink* (places)
*heavy*, *light* (weight)	*wool*, *cotton*, *silk* (materials)
	*rock*, *stick*, *feather* (objects)
*long*, *short* (duration/length)	*slacks*, *capris*, *shorts* (pants)
	*novel*, *story*, *poem* (readings)
*loud*, *quiet* (loudness)	*baby*, *teenager*, *adult* (people)
	*auditorium*, *classroom*, *study hall* (rooms)
*noisy*, *quiet* (loudness)	*horn*, *guitar*, *harp* (instruments)
	*powerboat*, *sailboat*, *row boat* (boats)
*light*, *dark* (luminance/color)	*day*, *dusk*, *night* (times of day)
	*white paint*, *blue paint*, *black paint* (paints)
*fast*, *slow* (speed)	*runner*, *skier*, *weight lifter* (athletes)
	*glider*, *helicopter*, *plane* (aircraft)
*quick*, *slow* (speed)	*rabbit*, *cat*, *turtle* (pets)
	*instant pot*, *frying pan*, *crockpot* (cookware)
*strong*, *weak* (strength)	*hurricane*, *thunderstorm*, *rain* (storms)
	*lion*, *dog*, *mouse* (animals)
*hard*, *soft* (hardness)	*jolly rancher*, *chocolate*, *marshmallow* (sweets)
	*tile*, *wood*, *carpet* (floor materials)
*wide*, *narrow* (width)	*boulevard*, *street*, *country lane* (roads)
	*truck*, *car*, *golf cart* (vehicles)

Each of the 90 item sets contains three relatively subordinate categories, which vary according to common‐sense, general expectations given by background knowledge about a degree (or, property value). For example, a basketball player, a soccer player, and a jockey vary according to common‐sense expectations about their relative heights (i.e., these categories fall on the high‐, medium‐, and low‐ends of the height scale, respectively). Furthermore, each set of three subordinate categories falls under the same relatively superordinate category (e.g., basketball players, soccer players, and jockeys are all *people* [the superordinate category]).^7^ The categories within each item set are then paired with positive‐form and negative‐form adjectives (e.g., tall and short). Thus, this experiment had 270 unique categories (90 sets × 3 subordinate categories of different general expectations/background knowledge) predicated in sentences by two adjectives each (e.g., tall and short), for a total of 540 unique items.

#### 3.1.3 Procedure

The experiment began with a warm‐up trial that was designed to convey the intuition behind comparison class inference. Participants were given the example of *John says: “The Empire State Building is tall”* and asked *What do you think John meant?*; participants responded by filling‐in a sentence in which the comparison class was made explicit (i.e., *The Empire State Building is tall relative to other ___*). Invalid responses to this warm‐up trial were used as a basis for exclusion (see the SI).

Participants then completed 36 main trials. Each main trial began with a *context sentence* that introduced the referent as a member of a subordinate category and provided an appropriate, minimal context in which the adjective could be uttered (e.g., *John sees a { basketball player, golfer, jockey}*); the same context sentence was used for all three categories in an item set. Then, a speaker utters an adjectival utterance predicating the adjective of a pronoun used to refer to the referent (e.g., *Mary says: “They're tall”*).^8^ The participant was asked what they thought the speaker meant (e.g., *What do you think Mary meant?*). Participants responded by freely filling in a sentence that required an explicit comparison class:

They're tall relative to other ______.

We used the word “other” to invoke the presupposition that the referent is a member of the comparison class. Pilot testing suggesting that omitting this word invoked many direct comparisons to singular entities (e.g., *They're tall relative to their short friend*), which were wildly heterogeneous in nature. After the main comparison class inference trials, participants completed a memory check trial where they had to select (from a checklist of 10 options) all of the adjective–noun phrase pairs (e.g., *tall – basketball player*, *green – tennis ball*) that they had seen on the main trials (see the SI for full details).

### 3.2 Results

Participants were excluded if they either responded incorrectly to the warm‐up trial (answering something other than *buildings*, *skyscrapers*, *structures*, or the like) or answered fewer than 7 out of 10 post‐test memory check questions accurately. Seven‐hundred fifty participants (89.6%) remained after these exclusion criteria, for a total of 27,000 responses. We additionally excluded nonsensical responses (443 in total, or 1.6%) and responses that resulted from the participant inferring an unintended referent for the pronoun in the target sentence (e.g., given “Alex is in a forest and hears a woodpecker. He says: *It is loud*,” the participant infers *it* refers to the forest; 267 in total, or 1.0%). We preprocessed the remaining 26,317 responses by correcting for misspellings (462; 1.8%), lemmatizing (3,823; 14.5%), and collapsing across synonyms (660; 2.5%; see the SI for details).

Pilot testing suggested that participants primarily provided comparison class paraphrases that were either identical to the subordinate noun phrase (NP) by which the referent was introduced (*subordinate‐NP*, e.g., basketball players) or a more superordinate category label (*superordinate‐NP*, e.g., people). We took advantage of the fact that conveying a category at least as specific as the subordinate‐NP generally requires including the subordinate‐NP in the response (e.g., *male basketball players* is more specific than *basketball players* and includes the substring *basketball player*)^9^ to automatically categorize responses as *subordinate* if the preprocessed response contained the subordinate‐NP as a substring (15,721, or 59.7% of all responses); all other responses were coded as *superordinate* (10,596, or 40.3%). Thus, “superordinate” here refers to categories potentially at different levels of abstraction (e.g., a wrestler may be strong relative to other *wrestlers*, *athletes*, or *people*; in this case, both *athletes* and *people* would be categorized as superordinate) as well as categories along different conceptual hierarchies (e.g., the price of a particular *top‐shelf liquor* could be expensive relative to other *alcoholic drinks* or relative to the same kind of top‐shelf liquor at *other stores*; a *pet parrot* could be loud relative to *other pets* or relative to other *birds*; etc.).

To better understand the distribution of free responses, we performed a secondary analysis where we first categorized the superordinate responses by whether or not they contained the modal superordinate‐NPs for that item.^10^ Of the 10,596 responses categorized as “superordinate,” 6,645 (25.2% of all responses) explicitly mention the modal superordinate‐NP for that item and 3,951 (15.0% of all responses) mention neither the subordinate nor the modal superordinate‐NP (“other” responses). Of the 15% of responses that mentioned neither subordinate nor superordinate responses, 77% of them (3,034 responses) were responses that were produced by at least three participants, suggesting that these were robust alternatives to the other more salient options (i.e., subordinate or modal superordinate). These “other” responses fell into five natural categories, in roughly equal proportions: categories at an intermediate level of abstraction between subordinate and superordinate (e.g., *melons* is intermediate between *watermelons* and *fruit*; *drinks* is intermediate between *smoothies* and *food*; 28%), categories that were superordinate to the modal superordinate response (e.g., *desserts* is superordinate to *cakes* which is superordinate to *cheesecakes*; *instruments* is superordinate to *guitars* which is superordinate to *ukeleles*; 28%), categories that were superordinate along different conceptual hierarchies than the modal superordinate category (e.g., chicken at a butcher shop that was expensive relative to other *butcher shops* as opposed to relative to other *meats*; the nighttime that was dark relative to other *places* as opposed to relative to other *times of day*; 14%), rough synonyms of the modal superordinate category (e.g., *bouquets* instead of *flower bouquets*; *methods of cooking* instead of *cookware*; 21% of “other” responses), and responses that referred directly to the scale or dimension described by the adjective, which could be construed as the most superordinate category (e.g., *expensive* relative to other *prices*; 9%). In the subsequent analyses, we treat the *other* responses that mention neither the subordinate or superordinate‐NP as *superordinate*. The qualitative results do not change, however, when we exclude these *other* responses altogether (see the SI for a parallel set of regression results excluding the *other* responses).

#### 3.2.1 Regression analysis

Our first test is to examine how subordinate versus superordinate comparison class inferences are influenced as a function of general expectations (i.e., background knowledge) about the category of the referent and the polarity of the adjective used to describe the referent. Specifically, we test for the qualitative pattern predicted by the comparison class inference model: an interaction between the general expectations about the category and the polarity of the adjective, which we tested with a maximal Bayesian mixed‐effects model.^11^ Further, the directionality of these effects arbitrates between the pragmatic versus literal comparison class inference models.

**Fig 3:**
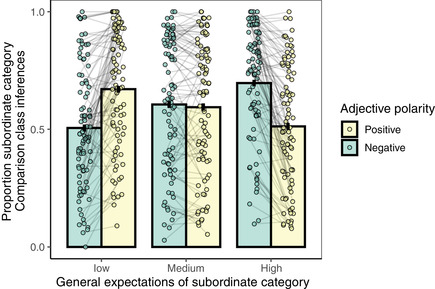
Comparison class inference experimental results. Proportion of paraphrases that contained the subordinate NPs (e.g., *basketball player*) with which the referent was introduced, as a function of the general expectations (background knowledge) listeners have about the category (*x*‐axis; e.g., gymnasts = low, basketball players = high) and the polarity of the adjective used to describe the category (color; e.g., *tall* = positive, *short* = negative). Bars represent overall means, and error bar is a bootstrapped 95% confidence interval. Each dot represents the mean of a single item and lines connect subordinate NPs described with different adjectives (e.g., *tall* and *short* basketball player). Dots are jittered horizontally to improve visual clarity.

As predicted by the comparison class inference model, we observe a pair of two‐way interactions in the directions predicted by the pragmatic, but not the literal, listener model (Fig. 3): When the subordinate category was expected to be near the high end of the scale (e.g., basketball player), the positive‐form adjective (e.g., tall) led to fewer subordinate comparison classes than the negative‐form adjective (e.g., short) in comparison to the control, middle‐of‐the‐scale items (e.g., soccer player): posterior mean beta‐weight and 95% Bayesian credible interval: β=−1.34[−1.67,−1.02]. This interaction was the result of the high‐end‐of‐the‐scale subordinate categories showing more subordinate comparison class inferences for the negative adjective (e.g., short for a basketball player) than for the positive adjective (e.g., tall for a basketball player): β=1.41[1.12,1.70]. No preference was observed for the middle‐of‐the‐scale, control items (e.g., tall for a soccer player vs. short for a soccer player; β = −0.06 [−0.24, 0.11]). A comparable interaction was observed for categories that were expected to be near the low‐end of the scale (e.g., jockey): Hearing the positive‐form adjective led to credibly more subordinate inferences (e.g., tall for a jockey) than hearing a negative‐form adjective (e.g., short for a jockey), in comparison to the middle‐of‐the‐scale subordinate categories: β=1.27[0.96,1.58]. Again, this effect was driven by the behavior of the low‐end‐of‐the‐scale subordinate categories, which showed a stronger preference for the superordinate comparison classes for negative than for positive adjectives (β=−1.20[−1.51,−0.90]).

Our results are remarkably symmetric across the low‐end‐ vs. high‐end‐of‐the‐scale subordinate categories. We observe an overall preference for subordinate comparison classes for the control (middle‐of‐the‐scale) subordinate categories (e.g., *soccer players*; β=0.71[0.32,1.10]) and no overall differences in this preference for items at the high‐end of the scale (e.g., *basketball players*; β=0.10[−0.19,0.41]) or the low‐end of the scale (e.g., *gymnasts*; β=0.02[−0.26,0.30]). The inferences that result from adjectives that are in conflict with a listener's general expectations of the categories (e.g., tall gymnasts vs. short basketball players) were not different between the low‐end and high‐end items (β=−0.18[−0.56,0.20]) nor were the inferences from adjectives that were consistent with general expectations about a category (e.g., short gymnasts vs. tall basketball players; β=0.02[−0.37,0.41]).

**Fig 4:**
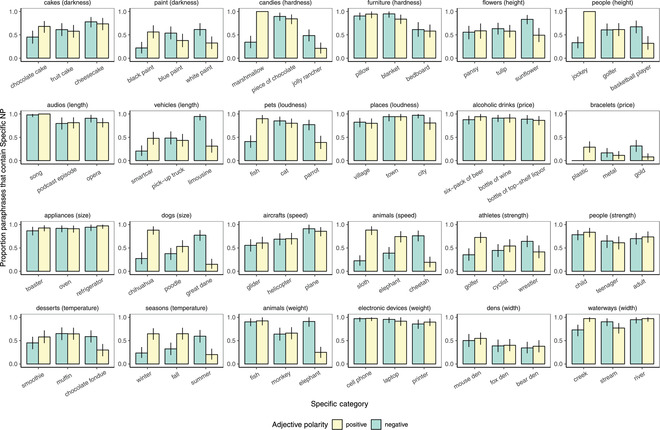
Comparison class inference results for 24 of the 90 item sets. Two examples were selected from each unique degree scale; example items were those that exhibited the greatest and smallest variability in comparison class inferences.

Taken together, these results point to comparison class inferences that are guided by general expectations given by background knowledge about the category and the adjective that a speaker uses to describe the referent, as predicted by the pragmatic model. Human listeners infer the most likely reference class given what their knowledge of the world tells them about the observations that would be remarkable enough for a speaker to mention. The overall preference for a subordinate‐level comparison class and the magnitude of the effect of the adjective varies considerably by item, however (Fig. 4). We next turn to the quantitative aspect of our model to triangulate the sources of this variability.

### 3.3 Quantitative modeling of item variability

#### 3.3.1 Modeling approach

Our model is not only useful for understanding the basic computations that underly comparison class inference. It is a tool for characterizing, by way of the gradience in the model's predictions, the subtleties of comparison class inference. The model's primary capacity to predict gradience in comparison class inference is as a function of background knowledge (or, *general expectations*) about properties of categories P(x) (e.g., the heights of basketball players, temperatures in Winter, etc.). Beyond knowledge about properties of categories, baseline expectations about what comparison classes speakers are likely to use P(c∣k) can also influence the model's predictions and be a source of variance in inferences. For the conceptual comparison classes that we focus on here, baseline expectations could vary by the level of abstraction of the categories in question (e.g., exhibiting a basic‐level bias; Rosch & Mervis, 1975) as well as the usage frequency of the NPs used to describe those categories. We thus construct a family of alternative models in order to gain insight into the sources of variability in comparison class inferences; these models differ in their parameterization of the comparison class prior: (1) a flat prior model, which assumes the comparison class prior assigns equal probability between subordinate and superordinate classes; (2) a basic‐level bias (or, intercept only) model, which assumes an a priori preference for basic‐level categories; (3) a frequency effect (or, slope only) model, which assumes that NPs that are used more frequently are more preferred as comparison classes, but without a basic‐level bias; (4) a basic‐level and frequency (or, slope and intercept) model, which assumes both a basic‐level bias and an effect of usage frequency.^12^ We estimate the distributional knowledge about properties P(x), the parameters of the comparison class prior (model‐variant dependent), and the speaker optimality parameter α (of the speaker model in Eq. 3) using Bayesian data analysis.

Since we infer the world knowledge priors P(x), our analysis technique is similar to a *descriptive Bayesian approach* (Tauber, Navarro, Perfors, & Steyvers, 2017), in which the modeler is simply interested in estimating what the prior knowledge in the model would have to be in order to account for the inference patterns in the data. Here, however, we go beyond merely accommodating the item‐level variation in the data and attempt to predict the variability in the data using independent measurements of participants' prior knowledge of categories and properties. Measuring prior knowledge in this setting is challenging, however, since direct empirical measurements require explicit estimation of relevant quantities or probabilities (e.g., plausible temperatures of days in winter, plausible heights of basketball players, etc.; Franke et al., 2016); while many of our domains can be reasoned about intuitively (e.g., the loudness of a diesel engine vs. an electric car), most people probably do not have access to a precise representation of the underlying scale (e.g., how many decibels is the typical sound of a diesel engine?). For this reason, we take a different approach from a canonical prior elicitation task: We take advantage of the fact that our computational model for comparison class inference includes a submodel for understanding scalar adjectives with explicit comparison classes (e.g., interpreting *an electric car is quiet relative to other cars*) and we use this submodel to simultaneously predict data from a separate experiment about these similar adjectival sentences. In this *adjective endorsement* task, a separate group of participants (n=375) provide truth judgments for adjectives with explicit comparison classes (e.g., “Imagine a day in Winter. Do you think it is cold relative to other days of the year?”; see the SI for details). We then use a joint Bayesian data analytic strategy to estimate a single set of parameters for the prior knowledge (e.g., the distribution of temperatures for days in Winter) which is shared between the two tasks.

We infer the parameters for all models using a Bayesian data analytic model that shares the world knowledge parameters between the two tasks (comparison class inference and adjective endorsements) and infers the parameters for the comparison class prior (details of which depend on the model variant: basic‐level bias, frequency effect, etc) and the speaker optimality free parameters of the RSA models (SI Figure S3). We implemented the RSA and Bayesian data analysis models in the probabilistic programming language WebPPL (Goodman & Stuhlmüller, 2014) and performed inference by running seven Markov chain Monte Carlo (MCMC) chains with 500,000 iterations each, discarding the first 250,000 for burn‐in. Convergence was checked through visual inspection of the different chains to ensure similar conclusions would be drawn from each chain independently.

#### 3.3.2 Model results

**Fig 5:**
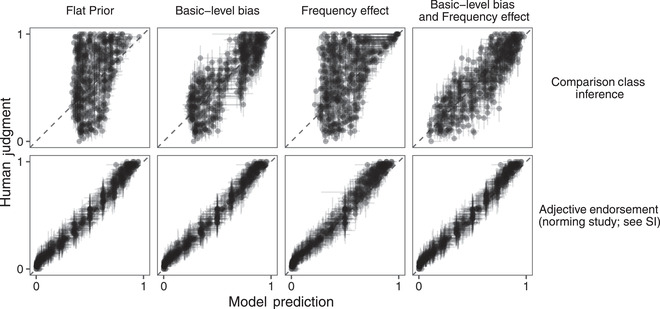
Model fits for main experiment (comparison class inference; top row) and norming experiment (adjective endorsement; bottom row) for four models that differ in their parameterization of the comparison class prior used inside the comparison class inference RSA model. The flat prior model assumes all comparison classes are equally likely a priori. The basic‐level bias model assumes that there is a preference for a basic‐level comparison class. The frequency effect assumes the prior probability of a comparison class tracks the frequency of the NP in a corpus. Basic‐level and frequency effect assumes that the prior probability of a comparison class is a function of a basic‐level bias and frequency. Dots represents means of the human judgments (proportion of subordinate‐NP responses for comparison class inference experiment; proportion endorsement for norming study) and the maximum a posteriori estimate of the model's predictions. Lines represent bootstrapped 95% confidence intervals for the data and 95% Bayesian credible intervals for the models.

**Fig 6:**
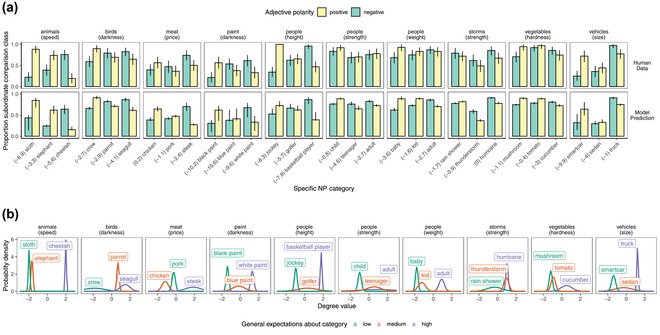
Quantitative modeling results for 10 sets of items. (See SI for full data‐analytic model.) A: Human comparison class inference data and model predictions for ten items. The log ratio corpus frequency of subordinate NP to superordinate NP (used in models with frequency‐effect) is shown in brackets next to the x‐axis labels; a more negative number corresponds to a stronger prior belief in the superordinate category as the comparison class. B: Imputed prior distributions over degrees for ten items. Distributions were generated from the Maximum A‐Posteriori parameter values inferred by conditioning on the Comparison Class Inference and Adjective Endorsement data sets. Superordinate‐level category distributions (e.g., heights of people) are assumed to be Unit Normal distributions for all item sets.

All model variants were able to accommodate the adjective endorsement dataset well (Fig. 5, bottom row). This result is an important sanity check; it shows that the parameters for world knowledge learned by the model are those that maximize the fit to the adjective endorsement data, and thus reflect this common‐sense world knowledge that this task taps into. Thus, the adjective endorsement task works as a kind of vague, prior elicitation, where we can learn about the world‐knowledge priors by asking simple natural language queries to participants. Correspondingly, the imputed world knowledge priors inferred by the Bayesian data analysis model reflect intuitively reasonable general expectations about the categories (e.g., the relative heights of basketball players vs. golfers vs. jockeys; Fig. 6b).

The predictions of the different model variants come apart for the comparison class inference data (Fig. 5; top row). The baseline flat prior model predicts variability in comparison class inferences only as a function of world knowledge about the properties, assuming all comparison classes are equally likely a priori; this model explains roughly 14% of the variance between items. The frequency effect model assumes NPs with higher usage frequency will more likely be used as comparison classes and is able to explain roughly 22% of the variance. The basic‐level bias model explains roughly 71% of the variance by assuming that basic‐level comparison classes may be more likely a priori. Finally, the maximally parameterized *basic‐level and frequency effect*—a combination of the two alternative models—gives rise to the best model predictions in terms of variance explained (77%) and mean squared error (Table 2), suggesting that the structure of the comparison class prior reflects both a basic‐level bias and an effect of the frequency of the NP, in addition to other possible factors. We confirm that the maximally parameterized model is the best explanation of the data by comparing the marginal likelihood of the data under each model to compute a Bayes factor (BF) as a measure of formal comparison. BFs quantify how well the model predicts the data, averaging over the prior distribution over parameters; by taking the average over the model's prior distribution over parameters, the measure explicitly takes into account model complexity because higher complexity models have wider prior distributions over parameters (Lee & Wagenmakers, 2014). The observed data are several orders of magnitude more likely under the full model than the closest competitor model, the simpler basic‐level bias only model (Table 2).

**Table 2:** Model evaluation results. Full basic‐level and frequency model exhibits the best fit to both datasets in terms of variance explained $\left(r^{2}\right)$ and mean squared error (MSE). (log) Bayes factor are shown with respect to the full model (i.e., negative numbers indicate positive evidence for the full basic‐level and frequency model)

Model	$r_{CC}^{2}$	$MSE_{CC}$	$r_{norming}^{2}$	$MSE_{norming}$	log BF
Flat prior	0.136	0.0712	0.987	0.0032	−2,569
Frequency effect	0.222	0.069	0.98	0.0058	−3,857
Basic‐level bias	0.715	0.0212	0.989	0.0028	−215
Basic‐level and frequency	0.769	0.0171	0.989	0.0028	0

**Fig 7:**
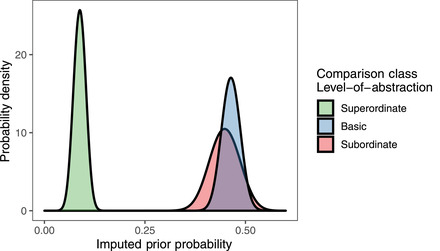
Imputed distributions over the prior probabilities of comparison classes at different levels of abstraction. Basic‐ and subordinate‐level categories comprise a priori likely comparison classes, while superordinate categories are less likely to serve as comparison classes.

To gain further insight into the comparison class inference results, we examine the maximal model's posterior distribution over parameters. The speaker optimality parameters for each task were inferred to be values consistent with the prior literature on RSA models—means and 95% Bayesian credible intervals: $\alpha_{1}=1.45\left(1.32,1.71\right)$ $\alpha_{2}=5.31\left(4.46,5.70\right)$. We also find a positive effect of frequency of the noun phrase—$\beta_{1}=0.21\left(0.09,0.21\right)$ —indicating that more frequent noun phrases give rise to more salient comparison classes. Our maximal model infers two parameters for the basic‐level bias, one which operates if the particular item is a subordinate‐level category (and by extension, the more superordinate category is a basic‐level category; $\beta_{0}^{0}=-0.10\left(-0.17,0.10\right))$ and one that operates if the item is a basic‐level category (and thus, the more superordinate category is a superordinate‐level category; $\beta_{0}^{1}=1.61\left(1.55,1.81\right))$. We can use these parameters to impute prior probabilities of subordinate, basic, and superordinate comparison classes (assuming a constant effect of usage frequency). These parameters show that basic and subordinate comparison classes are roughly equally accessible, while superordinate comparison classes are substantially less likely (Fig. 7).

Our fully parameterized model triangulates its inferences about the comparison class via background knowledge about the property and the comparison class prior, given by a basic‐level bias and the corpus frequency of the subordinate NP. Fig. 6 shows these sources of information for 10 sets of subordinate NPs. One can see that the middle‐of‐the‐scale control items were sometimes inferred to be closer to high‐end of the scale (e.g., the *parrot* is considered light in color relative to other birds) and sometimes closer to the low‐end of the scale (e.g., the *elephant* is considered slow relative to other animals; Fig. 6a). These judgments result in different imputed world knowledge priors (Fig. 6b), which leads to different comparison class inferences (Fig. 6a, comparison class inferences). For example, the *elephant* patterns more like the *sloth* than it does the *cheetah* in its comparison class inferences: when described as *fast*, it is more likely to be considered *fast for an elephant* than when it is described as *slow* (similar to the effect observed for *cheetah*).

The flexibility of the fully parameterized model allows it to make a wide range of quantitative predictions, which track the human data quite well. For example, comparing the *birds* items (Fig. 6a second column) to the *meat* items (Fig. 6a third column), we observe a dramatic difference in the overall preference for the subordinate versus superordinate comparison class (with the birds items more strongly preferring the subordinate categories, e.g., crow, parrot, seagull; and the meat items more strongly preferring the more superordinate category, e.g., meat), which can be accounted for by a stronger basic‐level bias for *birds* than for *meat*. The influence of corpus frequency in the comparison class prior can be seen most clearly for item sets where there are different baseline subordinate versus superordinate preferences within the same set of subordinate categories. For example, in the *vehicles* items (Fig. 6a right‐most column), we see a strong preference for the subordinate comparison class *trucks* in the human data; at the same time, the baseline preference is stronger for the superordinate *vehicles* when talking about *smart cars* or *sedans*. The model is able to capture this pattern through the frequency component of the prior on comparison classes (the log‐frequencies are shown in parentheses next to the category label in Fig. 6a); *trucks* is a more common label than either *smart cars* or *sedans*, which increases the model's baseline preference for the trucks comparison class.

The data on comparison class inferences, however, sometimes exhibits even more variability and sometimes exhibits less variability than predicted by our model. For example, the model predicts variability in the inferences from the *weak* versus *strong* *teenagers* versus *adults*, where no such variability is observed empirically (similarly for the entire item set of *spices*, see SI Fig. S7). Comparison classes inferences concerning the strengths of different walls (tents, condos, mansions) is largely a result of different baseline preferences: *tents* is a rather good comparison class whereas *condos* and *mansions* are less strongly preferred (SI Fig. S7). The variability for writing tools is also quite idiosyncratic: *pens* are largely compared to other *colors* (coded as superordinate), whereas *pencils* and *chalk* are compared to other pencils and chalk, respectively, as well as different *scripts* (superordinate). Taken together, these results indicate that human comparison class inferences are highly flexible, in a way that reflects distributional knowledge about properties of categories, a bias for basic‐level categories, and knowledge of usage frequency; still, further variance remains to be explained even in the tightly‐controlled experimental dataset we collected.

## 4 General discussion

Understanding the meanings of words often, if not always, requires appreciating the context in which those words are uttered. Yet, context is almost never articulated explicitly, but left to the listener to pragmatically reconstruct. Inferring comparison classes for relative statements (e.g., scalar adjectives like *tall*) is a case study in the larger problem of pragmatic reconstruction of context and is the communicative variant of the well‐known *reference class problem* in philosophy. In this paper, we propose that listeners flexibly adjust the comparison class by using their knowledge of the world to inform them about the kinds of observations that would be remarkable enough for a speaker to mention. We implement this proposal by extending an existing language understanding model to reason about comparison classes. Its prediction is empirically confirmed in an open‐ended response measure using a diverse range of linguistic stimuli. The strong fit of this model suggests that comparison class inference can be viewed as cooperative pragmatic inference.

We proposed a minimal extension to an adjective‐interpretation Rational Speech Act model to allow it to flexibly reason about the implicit comparison class (e.g., *tall for a person* vs. *tall for a basketball player*). We have thus addressed a particular aspect of comparison class inference: deciding among two conceptually‐based comparison classes, that we pre‐specified as subordinate versus superordinate category labels. The restriction of our model to consider only two comparison classes was a convenient simplification, but the fact that the majority of responses naturally produced in a free‐production experiment were either subordinate or superordinate category labels suggests that this was also a reasonable simplification. At the same time, the variability in responses was substantially greater than our idealized model could account for: Participants freely produced comparison classes that were either more abstract or less abstract than our superordinate comparison classes as well as comparison classes that fell along different conceptual hierarchies. The comparison class prior in our model could be elaborated to encode a more structured theory about how the hypothesis space of comparison classes is constructed (e.g., via a rational clustering model that considers higher‐order structures in categories and properties a la Kemp, Shafto, & Tenenbaum, 2012).

Going beyond general conceptual knowledge, many of the “miscellaneous” comparison classes that were elicited in our task appealed to other aspects of the context that were made available by the language in the task. For example, the sentence *Robert is at a decoration shop and looks at a statue made of bronze* brings into the context different concepts that could be parametrically combined to construct comparison classes—bronze, statues, decorations, shop—and several participants appealed to these concepts in their responses (e.g., expensive relative to *items at the shop*). These observations dovetail with prior work that investigated adjective interpretation with comparison classes in young children and which highlight the importance of linguistic cues for comparison classes. For example, for a 4‐year‐old, the comparison class for a “tall pimwit” is *other pimwits* and does not include other non‐pimwit objects (Barner & Snedeker, 2008). Even younger children can flexibly shift between qualitatively different kinds of comparison classes, given strong linguistic cues to distinguish the intended comparison class (e.g., “Is this a *big mitten*?” vs. “Is this mitten *big for the doll*?”; Ebeling & Gelman, 1994). One key future direction then is determining the cues that are available in the naturalistic environment and how adult and child listeners use those cues to infer comparison classes. The computational machinery we present in our model of comparison class inference is general and should apply equally well to these other kinds of comparison classes, once a hypothesis space of comparison classes is determined.

In addition to the novel empirical data and the computational model of comparison class inference, this paper presents experimental and data‐analytic methodological innovations. On the experimental side, we articulated an explicit generative model for our experimental stimuli, which we deployed on human participants to construct a large and diverse set of linguistic items (*n* = 540 unique stimuli). While this procedure was not entirely “end‐to‐end” (i.e., we authors still needed to curate, edit, and add context to the items), the method presents a significant advance beyond the traditional method of constructing a small set of stimuli, often inadvertently optimized to test a theory (Clark, 1973). On the data‐analytic side, we coupled a *descriptive Bayesian* approach (Tauber et al., 2017) with the productivity of probabilistic models of language understanding (Goodman & Frank, 2016; Scontras et al., 2018, 2021) to jointly model two complementary language tasks and infer the relevant prior knowledge shared between the tasks. The major feature of this method is that it allows us to back out quantitatively detailed domain knowledge that would otherwise be inaccessible through traditional prior elicitation techniques because human participants lack requisite knowledge of the quantitative scales (e.g., how many decibels is the sound of a rooster's crow?); in addition, this method has the feature that participants respond only to simple, natural language questions rather than estimate numerical quantities for which complicated linking functions must be designed (cf., Franke et al., 2016). This fully Bayesian language approach provides further constraints on the language understanding models, which must predict quantitative data from two related language experiments. This approach highlights how the productivity of natural language can be harnessed to productively design experiments to further constrain and test computational models of language and cognition.

In our stimuli, a speaker utters an adjectival sentence without a strong cue to the comparison class: *He [a basketball player] is tall*. Why might speakers not bother articulating the comparison class? If a listener is trying to infer the comparison class (like the model we present in this paper), then a speaker (who is reasoning about this listener) could save the effort of producing the comparison class when they believe the listener will correctly infer it.^13^ In the SI, we formalize this kind of higher‐order speaker, who reasons about whether or not the listener will correctly infer the comparison class; furthermore, we show that a higher‐order listener who reasons about this speaker draws qualitatively similar comparison class inferences to the simpler listener model we present above. Future work can be done to interrogate the predictions of these more sophisticated speaker and listener modelsto better understand when human speakers decide when to make the comparison class explicit and the inferences listeners may draw from that.

Comparison classes are useful for learning linguistic meanings because they allow learners to generalize the lexical semantics of many different kinds of adjectives and other linguistic messages that convey relative meanings. For example, the semantic meanings of *tall* and *warm* have equivalent semantic functional forms and vary only in the dimension the adjective picks up: [[tall]]=height(x)>θ and [[warm]]=temperature(x)>θ. Even generic language (e.g., *Birds have hollow bones*) can be understood by a threshold semantics: [[*Birds fly south in the winter*]] =P(xfliessouthinthewinter∣xisabird)>θ (Tessler & Goodman, 2019). Human cognition can use the minimal semantic template—[[words]]=dimension(x)>θ—and infer the intended comparison class (which determines θ) to create an infinity of possible meanings, and natural language seems to embrace these kinds of ambiguities (Piantadosi, Tily, & Gibson, 2012). It remains an open question, however, whether learning this common structural form of meaning requires a model with a built‐in structure. For example, large‐scale deep learning (especially transformer‐based) sequence models that are trained from huge amounts of text (i.e., large language models, such as the Bidirectional Encoder Representation from Transformers, or BERT, or the Generative Pre‐trained Transformers 3, or GPT‐3; Brown et al., 2020; Devlin, Chang, Lee, & Toutanova, 2018) exhibit surprisingly sophisticated syntactic knowledge, even on syntactic constructions that are rare in natural text (Futrell, Wilcox, Morita, & Levy, 2018; Linzen, Dupoux, & Goldberg, 2016; Wilcox, Futrell, & Levy, 2021). An important future direction for this line of work is to test whether such models learn a shared semantic representation for scalar adjectives (or, relative statements more broadly) and whether the large language models appreciate the context sensitivity that comes with different comparison classes.

The phenomenon of comparison class inference is a case study in *context inference*, or reasoning about the set of beliefs that are in common ground between speaker and listener. The model is thus similar in structure to models of presupposition accommodation (e.g., Degen, Tessler, & Goodman, 2015; Qing, Goodman, & Lassiter, 2016), which linguistic theories classically pose as involving a listener adding to or revising information from the common ground in order to make sense of an utterance. For example, if John says to Mary “My car is in the shop,” it is not necessary a priori that Mary knows that John has a car; if Mary did not know (or she temporarily forgot) that John owns a car, Mary can *accommodate* John's utterance by adding to the common ground the presupposition that *John has a car*. The continuity in the computational formalisms that underly this sort of common ground revision suggest a very basic cognitive mechanism behind the pragmatic reconstruction of context (e.g., Levinson, 2000).

Comparison class inference is also related to the well‐known problem of *reference classes* (Hájek, 2007; Reichenbach, 1949). In communication, pragmatic mechanisms are at play in how listeners decide upon an appropriate comparison class. This investigation thus raises the question of whether or not similar social reasoning occurs in legal or actuarial contexts, where reference class inference can impact everything from the value of a house to the severity of a criminal sentence (Cheng, 2009; Colyvan et al., 2001). If so, the very way in which we appreciate justice being served or the value of property stems from a cooperative, communicative framework.

## 5 Conclusion

The words we say are often too vague to have a single, precise meaning and only make sense in context. Aspects of the context, however, can also be underspecified, leaving the listener in the dark about both the speaker's intended meaning and about the context through which the listener is to make sense of the conversation. This work suggests that listeners infer a lot from a little: Meaning and context from simple, vague utterances.

### Open Research Badges

This article has earned Open Data and Open Materials badges. Data are available at https://github.com/mhtess/comparison‐class‐paper/tree/master/data and materials are available at https://github.com/mhtess/comparison‐class‐paper/tree/master/experiments.

## Supporting information


**Fig** **ure S1**: Overview of Experimental Tasks.
**Figure S2**: Proportion of responses under a three‐way classification: mention subordinate, mention the modal superordinate response, or mention another, relatively superordinate category.
**Figure S3**: Comparison Class Inference experimental results removing responses that mention neither subordinate nor superordinate categories
**Table S1**: Counts of items that had 100% subordinate‐NP comparison class responses, as a function of the adjective polarity (positive vs. negative) and general expectations about the category (low, medium, high) computed over the full dataset.
**Table S2**: Counts of items that had 100% subordinate‐NP comparison class responses, as a function of the adjective polarity (positive vs. negative) and general expectations about the category (low, medium, high) computed over the restricted data set.
**Figure S4**: Adjective Endorsement (Task 3) experimental results
**Figure S5**: Task 3 (Adjective Endorsement) results for a subset of the items.
**Figure S6**. Alternative model predictions.
**Figure S7**. Joint Bayesian data analytic strategy of the maximal model.
**Figure S8**. Quantitative modeling results for eight sets of items which show the lowest and highest residuals for the comparison class inference data.

## Notes

1 The terms *reference classes* and *comparison classes* seemingly have independent lives in independent literatures, but they are extremely related concepts. Given that our work uses language as a way to gain insight into the reference class problem, we use the terminology *comparison classes*—familiar to the linguistics literature—throughout.

2 More generally, we can distinguish shared beliefs from private beliefs. The hypothesis space of comparison classes is constrained by the shared beliefs of the speaker and listener: A listener will not assume the speaker is using a comparison class about which the speaker is ignorant, and a listener cannot construct a comparison class from information that they do not have. By contrast, expectations about the degree value for the referent (e.g., the referent's height) will be guided by the totality of the listener's beliefs—both those shared with the speaker and those not shared, the listener's private beliefs. For example, if the listener had private knowledge that the referent in question was taking a growth‐stunting medication, they would use that knowledge to guide their expectations about that person's height. If the listener knew that the speaker did not know about the growth‐stunting medication, that information would not enter into the hypothesis space of comparison classes. Formally, this more general model would be given by

(2)
$$\begin{array}{cc} L_{1}\left(x,c|u,f,g\right) & \propto S_{1}\left(u|x,c\right)\cdot P\left(x|f,g\right)\cdot P\left(c|f\right), \end{array}$$

where f denotes the beliefs shared between speaker and listener and g denotes the listener's private beliefs. Since our paradigm does not distinguish between these two kinds of beliefs, we use a simplified form of the model, where beliefs are represented by a single variable k and can be thought of as the most specific relevant category information about the referent (e.g., the referent is a basketball player).

3 For simplicity, for all of our qualitative and quantitative modeling, we assume no difference in production cost for different utterances. Hence, our model reduces to simply: $S_{1}\left(u|x,c\right)\propto L_{0}{\left(x|u,c\right)}^{\alpha }$

4 The determination of the set of alternative utterances in a Rational Speech Act model is a degree of freedom on the side of the modeler. We think this set is simple and reasonable, and we note that the inferences we model are invariant to other reasonable choices of alternative utterances. Most notably, including alternatives that allow the speaker to explicitly communicate the comparison class (e.g., the speaker could have said *tall for a basketball player* or *tall for a person*) do not change the key qualitative patterns we describe.

5 Following standard semantic treatment of antonyms, the semantics of *short* are a threshold function on a distinct threshold variable: $\left[\;\left[u_{short}\right]\;\right]=height<\theta_{short}$), which is also inferred by the listener model (i.e., the listener infers a threshold for both *tall* and *short*). The pragmatic inferences about the comparison class that are the focus of this paper are invariant to whether or not the antonym is included in the alternative set. The comparison class inferences are also invariant to whether or not the antonym is assigned its own unique threshold ($\theta_{short}$). See Lassiter and Goodman (2017) and Tessler and Franke (2018) for an exploration of the implications of assigning antonyms their own thresholds versus negating the threshold of the positive adjective.

6 The treatment of the threshold inference at the level of the literal listener is a point of divergence from the model of Lassiter and Goodman (2013, 2017), who instead argue for “lifting” the threshold inference to the level of the pragmatic listener (i.e., the pragmatic listener infers what is likely to be an *informative threshold*, whereas the literal listener merely infers what is likely to be *true threshold*). The treatment of threshold inference at the level of the literal listener is consistent, however, with Tessler and Goodman (2019)'s model of generic language interpretation. The question of whether the threshold inference should be thought of as a pragmatic inference is an empirical question and one that is beyond the scope of this paper. The qualitative predictions of the model about the comparison class are similar if the threshold is lifted to the pragmatic listener in such a way that the pragmatic listener jointly reasons about the threshold θ, the degree x, and the comparison class c.

7 We refer to the level of abstraction of these categories as *relatively* subordinate or superordinate, because the item sets may be sets of (i) subordinate‐level categories within the same basic‐level category (e.g., Great Danes, chihuahuas, within dogs) or (ii) basic‐level categories within the same superordinate‐level category (e.g., elephants, squirrels, within animals). Later, we will explicitly model this distinction in possible levels of abstraction of these categories.

8 We had the speaker use a pronoun so as to not provide a strong linguistic cue as to the intended comparison class. If the referent was a person, we used the singular “they” to refer to them; otherwise, we used either “it” or “they” depending on the plurality of the referent.

9 Negation markers (e.g., “all kinds of people *except* basketball players”) are one case where this assumption would be invalid, but no responses used negation markers in this way.

10 We experimenters guessed the modal superordinate‐NPs for each item (e.g., shown in Table 1), and in 89 out of 90 cases, the pre‐designated superordinate‐NP was the same the empirically most popular response. For the one item that our initial guess was incorrect (*desserts* for the set of *smoothies*, *muffins*, and *chocolate fondue*; the modal superordinate NP was in fact *foods*), we replaced our pre‐designated superordinate‐NP with the empirical modal superordinate‐NP (*foods*).

11 For the regression analysis, we predict subordinate‐NP paraphrases as a function of the general expectations about the subordinate category (low, medium, high; dummy coded with the medium category as the reference level), the adjective (positive vs. negative; difference coded), and their interaction; in addition, we include the maximal mixed effects structure by‐item set and by‐participant that mirrors this fixed effects structure. The model is subordinate_inference ∼ gen_expectations * adjective_polarity + (1 + gen_expectations * adjective_polarity | participant) + (1 + gen_expectations * adjective_polarity | item_set).

12 Because our stimuli were generated by (a separate group of) participants, we do not know a priori if the NPs we used in the task correspond to basic‐level or subordinate‐level categories (and, by extension, whether the relatively more superordinate class in our stimuli corresponds to a superordinate‐level or a basic‐level category). Further, a basic‐level bias could operate differently for a subordinate versus basic‐level inference than for a basic versus superordinate‐level inference. Thus, we endow our data‐analytic model with two regression coefficient parameters corresponding to different intercept terms (i.e., a basic‐level bias term for basic vs. subordinate categories and a basic‐level bias term for basic vs. superordinate categories), and we introduce a Bernoulli random variable z for each NP (each relatively subordinate category) to indicate whether it is a subordinate‐level term or basic‐level term. Further details can be found in the SI.

13 The speaker here would be operating roughly according to a principle of trying to communicate more information with less effort.
