# Supplementary material for: Warm (for Winter): Inferring Comparison Classes in Communication
Source: Cogn Sci. 2022 Mar 16;46(3):e13095. doi: 10.1111/cogs.13095 (PMC9286384; doi:10.1111/cogs.13095)
Supplement: Supplementary file 1 — Fig ure S1: Overview of Experimental Tasks. Figure S2: Proportion of responses under a three‐way classification: mention subordinate, mention the modal superordinate response, or mention another, relatively superordinate category. Figure S3: Comparison Class Inference experimental results removing responses that mention neither subordinate nor superordinate categories Table S1: Counts of items that had 100% subordinate‐NP comparison class responses, as a function of the adjective polarity (positive vs. negative) and general expectations about the category (low, medium, high) computed over the full dataset. Table S2: Counts of items that had 100% subordinate‐NP comparison class responses, as a function of the adjective polarity (positive vs. negative) and general expectations about the category (low, medium, high) computed over the restricted data set. Figure S4: Adjective Endorsement (Task 3) experimental results Figure S5: Task 3 (Adjective Endorsement) results for a subset of the items. Figure S6. Alternative model predictions. Figure S7. Joint Bayesian data analytic strategy of the maximal model. Figure S8. Quantitative modeling results for eight sets of items which show the lowest and highest residuals for the comparison class inference data. [file COGS-46-0-s001.pdf]

Supplemental Information
Warm (for Winter): Inferring comparison classes in communication

Michael Henry Tessler[1,2] and Noah D. Goodman[2, 3]

[1]Department of Brain and Cognitive Sciences, Massachusetts Institute of Technology
[2]Department of Psychology, Stanford University
[3]Department of Computer Science, Stanford University

Here we present additional information about methods, results, and cognitive models. Readers who are interested in the model code and analysis code are encouraged to consult the associated online repository: `https://github.com/mhtess/comparison-class-paper`.

Overview of Tasks

Our experimental procedures involve three coordinated experiments to (1) elicit test stimuli, (2) measure comparison class inferences and (3) measure adjective endorsements to facilitate model comparison (Figure S1). In Task 1, we empirically elicit test stimuli by having participants fill out phrasal templates that elicit sets of categories at the same level of abstraction, which differ in the general expectations participants have about those categories (e.g., basketball players [generally tall], jockeys [generally short], soccer players [sometimes tall and sometimes short]). From this set, we curate a set of experimental stimuli which we use in the other two tasks. Task 2 is the comparison class inference experiment described in the main text; this task is designed to test the qualitative predictions shown in Figure 1E (main text). Task 3 (an adjective endorsement, or truth judgment task) provides additional linguistic judgments that constrain model parameters to test the quantitative predictions of the comparison class inference model and quantitatively arbitrate between alternative models.

Experimental Methods

Bot check

In all tasks, participants were required to pass a simple language comprehension test that we designed in order to weed out bots and other bad-faith participants. The test involved a sentence in which a named speaker (e.g., Joseph) says to a named listener (e.g., Elizabeth) “It’s a beautiful day, isn’t it?”. Participants were asked to type in a text box to whom the speaker (in this case: Joseph) is talking (i.e., Elizabeth). Speaker and listener names were randomized in a way that could not be read off the source .html file. Participants

were given three attempts to correctly identify the listener. If they did not succeed within 3 attempts, they would be unable to proceed with the experiment. Since participants who fail this test are not allowed to proceed with the experiment, we do not have a count of how many participants fail this check.

Stimuli Generation Task (Task 1)

In this task, participants ($n = 50$) filled out phrasal templates for adjective pairs (e.g., *big* and *small*), in which three missing noun phrases which appeared in the grammatical subject of the sentence were described as either generally having one adjective apply to them (e.g., _____ *are generally big*; _____ *are generally small*) or as sometimes having either adjective apply to them (e.g., _____ *are sometimes big and sometimes small*). Participants filled out one template for each of 15 pairs of adjectives that describe physical dimensions (Table 1).

From this set of 750 responses, we curated a collection of 90 "item sets", each of which consists of 3 relatively subordinate level categories that differ in their general expectations about the degree (e.g., Winter, Spring, and Summer, which differ in their general expectations about the typical temperature). In addition, each of these item sets is associated with a common, relatively superordinate level categories (e.g., days of the year).

Comparison Class Inference (Task 2)

In this experiment, we measure comparison class inferences by having participants rephrase a speaker's statement which involves a scalar adjective in a way that makes the comparison class explicit. We measure comparison class inferences using a free-production measure to provide further ecological validity to our measurements of comparison classes: We wish to see if listeners spontaneously adjust their comparison class depending on world knowledge and pragmatic reasoning. A smaller-scale, forced-choice version of this task was reported in Anonymous (2017). Sample size, exclusion criteria, regression analysis, and cognitive model analysis were preregistered: `osf.io/xuc96`.

Participant restrictions and exclusion criteria. We recruited 837 participants from Amazon's Mechanical Turk. Participants were restricted to those with U.S. IP addresses with at least a 95% work approval rating. In addition, participants were required to pass a simple language comprehension test that we designed in order to weed out bots and other bad-faith participants, described above.

In addition to the above restrictions, we excluded participants based on both a task comprehension question appearing before the main trials and a memory check trial appearing after the main trials. In the task comprehension / warm-up trial, participants were told they they would be asked to rephrase something a person said: the person said a word that is relative and their task was to figure out what the word was relative to. They were given the example of *John says: "The Empire State Building is tall"* and asked to fill-in a sentence with the same kind of response they would do on the main trials (i.e., *The Empire State Building is tall relative to other* _____). Participants were told to fill in the blank with a group or category that makes the most sense and to use their common sense. Responses to this warm-up trial were used as a basis for exclusion (any response other than buildings, structures, towers, skyscrapers, or any misspellings thereof). Invalid responses

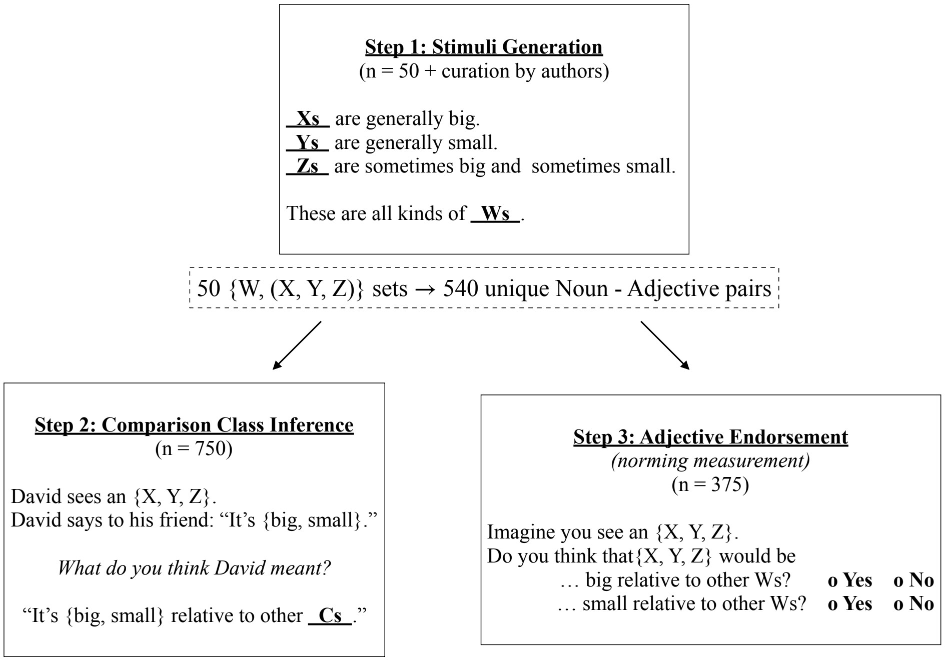

Figure S1. Overview of Experimental Tasks. Task 1: Using a structured production task, we elicit sets of stimuli that all share the feature of containing categories generally judged as having either a positive or negative adjective (X, Y; e.g., big or small) applied to them as well as a control category (Z; e.g., sometimes big and sometimes small). The task is designed in a way to elicit three categories of the same basic- or superordinate-level category (W). Task 2: Free-production task to elicit the comparison class. Task 3: Forced-choice task where participants judge whether a member of the subordinate level category would be judged as a having the adjective applied explicitly relative to the basic/superordinate level category. This task serves to provide additional data to constrain the parameters of the comparison class inference model.

to the warm-up trial were most often indicative of copying some part of the text on the screen and pasting it into the response box (e.g., responding with the name of the speaker, just putting the adjective "tall", or responding with a whole sentence without a comparison class "that is tall"). 55 participants were excluded for providing an invalid response to this task comprehension question.

After the main comparison class inference trials, participants completed a memory check trial asking which adjective–Noun Phrase (NP) combinations appeared on the main trials. At the end of the task, participants were asked a memory check question where they had to select, from a list of 10 options, all of the items they could recall seeing. In the memory check, items were shown as adjective – noun pairs ("tall – basketball player") and the 5 distractors were either color or multidimensional adjectives paired with a category that was not used in our test stimuli (e.g., "green – tennis ball"; "beautiful – painting"). Participants were excluded if they answered fewer than 7 out of 10 memory check questions correctly. A total of 59 participants failed this check, though 27 of these also failed the task comprehension check.

A total of 87 participants were excluded for meeting at least one of these exclusion

criteria, leaving a sample of 750 participants for the primary analyses.

Adaptive data collection procedure. Our experiment contains 540 unique items (adjective – NP pairs), which are highly heterogeneous. In addition to testing the main qualitative predictions of our models (adjective polarity X category expectations interactions), this experiment was designed to elicit high inter-item variability in comparison class inferneces. As a result, we expect some items to exhibit low intra-item variability (i.e., all or almost all participants respond with the same comparison class); for these items, we would require relatively fewer data points to estimate the parameter of interest – the probability of a subordinate vs. superordinate comparison class – since it would either be close to ceiling or close to floor.

We thus used a sequential sampling method that we deployed on an item-wise basis, wherein we paused data collection after collecting 35 responses for each of the 540 items. We then analyzed the partial data set on a by-item basis to see which (if any) items had received exceedingly consistent responses, which we defined to be at least 33 out of 35 ($>$ 94% agreement) of the same response. For those items that received exceedingly consistent responses, we stopped data collection at these 35 responses. We then continued collecting data on the items that received variable responses, until we had data from 750 participants after exclusion criteria were applied. This procedure allowed us to focus resources on providing better estimates for the items with more intra-item variability in responses. This adaptive procedure was decided ahead of time and is documented in the pre-registration report.

Response preprocessing. Our text preprocessing procedure was divided into two stages: Exclusions and Corrections.

Exclusions. We excluded responses that did not make sense given the context. These included but were not limited to responses that were simply the adjectives (e.g., *tall*), the names of the characters (e.g., *Alex*), and copied portions of the text on the screen (e.g., the sentence prompt: *the street is wide*). These invalid responses totaled 443 in total (1.6%).

We additionally removed from the data set responses that seemed to be the result of a failure of the participant to grasp the intended referent of the utterance. For example, in the context sentence: "Alexander is in the forest and hears a woodpecker. Alexander says, *It is loud*", the intended referent of the pronoun *it* is the woodpecker; however, some participants responded *it is loud relative to other forests*, which suggests they understood the referent to which *it* refers to be the forest. This kind of "reference failure" responses numbered 267 in total (or, 1.0% of the remaining responses).

Corrections. In order to improve alignment across responses, we corrected for misspellings (462; 1.8%), lemmatized to align plural and singular responses (e.g., *baby* and *babies*; 3823; 14.5%), and replaced some responses with obvious synonyms given the context (e.g., *child–kid*; *booze–alcoholic drink*; *humans/individuals–people*; 660; 2.5%).

Response analysis. After preprocessing the raw text responses, we preformed an automatic analysis of the comparison class responses by checking whether or not the preprocessed responses contained the subordinate NP presented in the experiment (e.g., *basketball player*) as a substring.

Pilot testing suggested that participants primarily provide comparison class paraphrases that are identical to the subordinate noun phrase by which the referent is introduced (*subordinate-NP*, e.g., a basketball player) or a more superordinate category (*superordinate-*

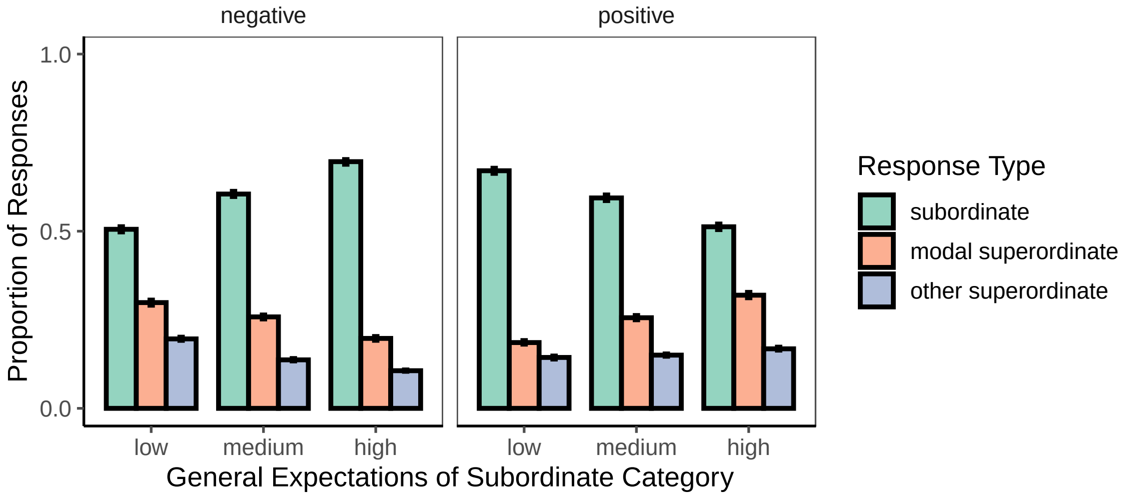

Figure S2. Proportion of responses under a three-way classification: mention subordinate, mention the modal superordinate response, or mention another, relatively superordinate category. For negative polarity adjectives, the proportion of both the "superordinate" and "other" responses increases as general expectations lower (and thus become more consistent with the adjective polarity; e.g., a short gymnast). For positive polarity adjectives, we see the reverse trend. Error-bars denote bootstrapped 95% CIs.

NP). In order to convey a category at least as specific as the subordinate-NP, one must include the subordinate-NP in the response (e.g., *male basketball players* is more specific than *basketball players* and includes the substring *basketball player*). One exception to this generality is utterances involving negation markers (e.g., "all kinds of people except basketball players"), but no responses in our data set involved negation in this way. Thus, we automatically categorized preprocessed responses as either subordinate or superordinate by checking whether the preprocessed response contained the subordinate-NP as a substring.

Supplementary data analysis.

Distribution of response types across experimental factors. In the main text, we reported that roughly 60% of responses explicitly mentioned the subordinate category, 25% of responses explicitly mentioned the modal superordinate category, and 15% of responses referred to some other, relatively superordinate category. We examine this 3-way classification of responses by the experimental factors that are the focus of this paper: general expectations about the category and the adjective polarity. Figure S2 shows the distribution of response types by general expectations and adjective polarity. Overall, we find a pattern of responses that is consistent with the idea that when the adjective is consistent with the general expectations about the category, a listener will look for a more superordinate comparison class.

Regression on subset of responses that explicitly mention subordinate or superordinate-NP. In the main text, we analyze the full data set of preprocessed responses. Here, we report statistics on a subset of the data set in which the participants preprocessed responses explicitly mention either the subordinate-NP (the NP by which the referent was introduced) or the superordinate-NP (an NP that the authors pre-specified as

the superordinate-NP for a set of three subordinate-NPs). 4007 responses (15.2%) are thus excluded from this analysis.

The results on the subset of responses that explicitly mention either the subordinate or superordinate-NP are entirely consistent with the results derived from the full data set. When the subordinate category was expected to be near the high-end of the scale (e.g., basketball player), the positive-form adjective (e.g., tall) led to fewer subordinate comparison classes than the negative-form adjective (e.g., short) in comparison to the control, middle-of-the-scale items (e.g., soccer player): posterior mean beta-weight and 95% Bayesian credible interval: $\beta = -1.51$ $[-1.94, -1.10]$. Again, this interaction was the result of the high-end-of-the-scale subordinate categories showing more subordinate comparison class inferences for the negative adjective (e.g., short) than for the positive adjective (e.g., tall): $\beta = 1.49$ $[1.12, 1.86]$, a preference which was not observed for the middle-of-the-scale, control items (e.g., *soccer players*; $\beta = 0.02$ $[-0.23, 0.28]$). As before, a comparable interaction was observed for categories that were expected to be near the low-end of the scale (e.g., jockey): Hearing the positive-form adjective led to credibly more subordinate inferences than hearing a negative-form adjective, in comparison to the middle-of-the-scale subordinate categories: $\beta = 1.42$ $[1.02, 1.83]$. Again, this was driven by the behavior of the low-end-of-the-scale subordinate categories, which showed a stronger preference for the superordinate comparison classes for negative than for positive adjectives ($\beta = -1.44$ $[-1.87, -1.04]$).

The inferences measured from this reduced data set are similarly highly symmetric across the low-end vs. high-end of the scale subordinate categories. We observe an overall preference for subordinate comparison classes for the control (middle-of-the-scale) subordinate categories (e.g., *soccer players*; $\beta = 1.68$ $[1.16, 2.21]$) and no overall differences in this preference for items at the high-end of the scale (e.g., *basketball players*; $\beta = 0.10$ $[-0.25, 0.44]$) or the low-end of the scale (e.g., *gymnasts*; $\beta = 0.23$ $[-0.15, 0.61]$). The inferences that result from adjectives that are in conflict with a listener's general expectations of the categories (e.g., tall gymnasts vs. short basketball players) were not different between the low-end and high-end items ($\beta = 0.11$ $[-0.42, 0.66]$), nor were the inferences from adjectives that were consistent with general expectations about a category (e.g., short gymnasts vs. tall basketball players; $\beta = 0.15$ $[-0.35, 0.65]$).

Universally consistent responses. 16 of our 540 items (adjective – noun pairs) had universally consistent lemmatized responses wherein all participants gave the same response; this number increases to 89 on the restricted data set that only includes responses that match either the subordinate or superordinate responses (described in the previous section). Of these universal consistent responses (i.e., responses that were at floor or at ceiling), all but one were cases where 100% of participants gave a subordinate-NP response (i.e., a response that was the same as the NP with which the referent was introduced). For example, 100% of valid responses for a "loud church" all concerned the subordinate category *church*. The lone NP-adj pair that led to all superordinate responses (for either data set) was the "cheap plastic bracelet", which all participants said was cheap relative to other *bracelets*. Intriguingly, the numeric counts of items that received 100% subordinate-NP responses mirrors the overall pattern of responses collapsed across items shown in the main text: adjectives that conflict with the general expectations about the category (e.g., a short basketball player) are more likely to receive subordinate-NP comparison class responses

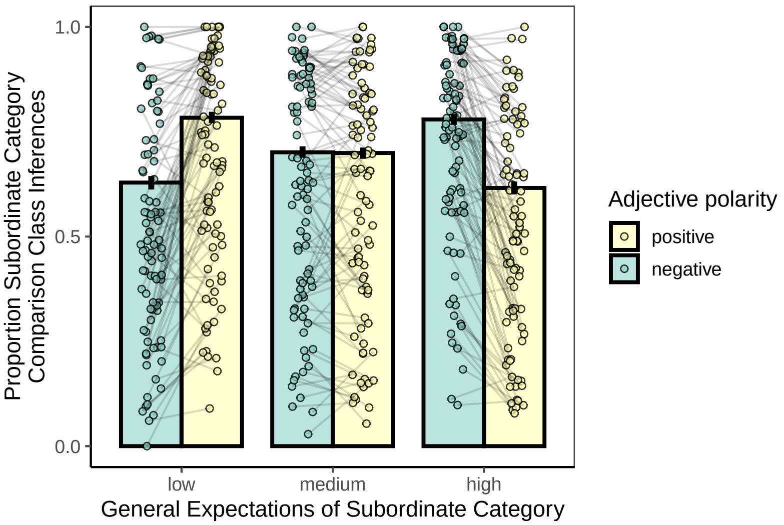

Figure S3. Comparison Class Inference experimental results removing responses that mention neither subordinate nor superordinate categories. Proportion of paraphrases that contained the Subordinate NPs (e.g., *basketball player*) with which the referent was introduced, as a function of the general expectations listeners have about the category (x-axis) and the polarity of the adjective used to describe the category (e.g., *tall* = positive, *short* = negative). Bars represent overall means and error bars are bootstrapped 95% confidence intervals. Each dot represents the mean of a single item and lines connect Subordinate NPs described with different adjectives (e.g., *tall* and *short* basketball player). Dots are jittered horizontally to improve visual clarity.

(Table S1 for counts of universally consistent responses from the full data set; Table S2 for universally consistent responses from the restricted data set).

Adjective Endorsement Task (Task 3)

This experiment served to generate further data that could be used to constrain the model parameters shown in Figure S7. Sample size, sampling procedure, exclusion criteria, and data analysis were preregistered: `osf.io/vdkbp`.

Participants. We recruited 400 participants from Amazon's Mechanical Turk, restricted to those with U.S. IP addresses with at least a 95% work approval rating. This number was arrived at with the goal of estimating endorsement probabilities for each item with a 95% confidence interval width of 0.2 for each item. Because our measure is a two-alternative forced-choice and because we expect some items to receive fairly categorical judgments (i.e., low between-participant variability), we employed an analogous sequential sampling procedure to that employed in Task 2 (Comparison Class Inference, see above) in order to over-sample items that exhibit high between-participant variability. We first collected 35 responses for each item, computed 95% CIs for each item, and stopped collecting data for items whose 95% CIs were smaller than 0.2. After the first 35 responses, we collected more responses for each item until the 95% CI for that item was smaller than 0.2 or we reached our predetermined sample size. The experiment took about 10 minutes and

Adjective Polarity	low	medium	high
negative	1	2	4
positive	7	0	1

Table S1
Counts of items that had 100% subordinate-NP comparison class responses, as a function of the adjective polarity (positive vs. negative) and general expectations about the category (low, medium, high) computed over the full data set.

Adjective Polarity	low	medium	high
negative	12	11	21
positive	23	15	7

Table S2
Counts of items that had 100% subordinate-NP comparison class responses, as a function of the adjective polarity (positive vs. negative) and general expectations about the category (low, medium, high) computed over the restricted data set.

participants were compensated $1.50 for their work.

Procedure. On each trial, participants were given a sentence introducing a member of a subordinate category (e.g., *You step outside during the winter.*). This was followed by a question asking if the participant would endorse the positive- and/or negative-form adjective explicitly relative to the superordinate category (e.g., *Do you think it would be warm relative to other days of the year? Do you think it would be cold relative to other days of the year?*). Both questions appeared on the screen on the same trial. Participants could respond to each question with either a *yes* or *no* judgment (2 judgments per trial). Each participant completed 48 items.

As with the comparison class inference experiment, after the main trials, participants completed a memory check trial asking which adjective–NP combinations appeared on the main trials. Participants were asked a memory check question where they had to select, from a list of 10 options, all of the items they could recall seeing. In the memory check, items were shown as adjective – noun pairs ("tall – basketball player") and the 5 distractors were either color or multidimensional adjectives paired with a category that was not used in our test stimuli (e.g., "green – tennis ball"; "beautiful – painting"). Participants were excluded if they answered fewer than 7 out of 10 memory check questions correctly. A total of 103 participants failed this check, leaving 297 participants for the main analysis.

Materials. The experimental materials were the same as used in Task 2 (Comparison Class Inference). Each trial contained 2 judgments (positive and negative adjectives).

Results. The primary goal of this experiment is to validate the stimuli generated in Task 1 and to use to data to constrain the parameters governing world knowledge in the joint Bayesian data-analytic model. We build a Bayesian logistic mixed-effects model to predict participants' responses as a function of the general expectations about the subordinate category (low, medium, high; dummy coded with the medium category as the reference level), the adjective (positive vs. negative; difference coded), and their interaction; in addition, we include the maximal mixed effects structure by-item set and by-participant that mirrors this fixed effects structure.[1]

The pattern of endorsements was highly consistent with the a priori general expectations by which the stimuli were generated (Figure S4). When the subordinate category was

[1] The model is judgment ~ gen_expectations * adjective_polarity + (1 + gen_expectations * adjective_polarity | participant) + (1 + gen_expectations * adjective_polarity | item_set).

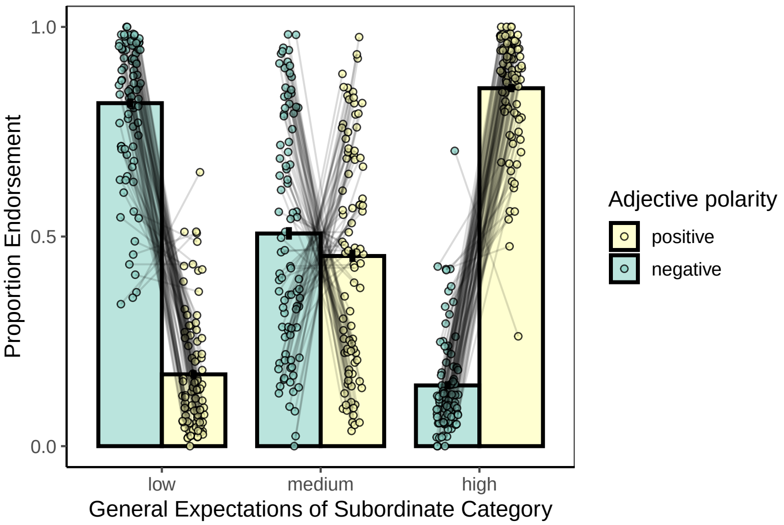

Figure S4. Adjective Endorsement (Task 3) experimental results. Proportion of sentence endorsements as a function of the general expectations listeners have about the category (x-axis) and the polarity of the adjective used to describe the category (colors; e.g., *tall* = positive, *short* = negative). Bars represent overall means and error bar is a bootstrapped 95% confidence interval. Each dot represents the mean of a single item and lines connect Subordinate NPs described with different adjectives (e.g., *tall* and *short* basketball player). Dots are jittered horizontally to improve visual clarity. The biggest outlier for the low general expectations was the loudness of the *owl*, which was judged to be more loud than quiet; the biggest outlier for the high general expectations was the fabric of *wool*, which was judged to be more light than heavy.

expected to be near the high-end of the scale (e.g., basketball player), the positive-form adjective (e.g., tall) was endorsed much more strongly than the negative adjective (e.g., short) in comparison to the control, middle-of-the-scale items (e.g., soccer player): posterior mean beta-weight and 95% Bayesian credible interval: $\beta = 5.72$ [4.81, 6.64]. This interaction was the result of the high-end-of-the-scale subordinate categories showing more endorsements for the positive (e.g., tall) than for the negative (e.g., short) adjective: $\beta = -5.38$ $[-6.02, -4.75]$. The same endorsement profile was not observed for the middle-of-the-scale, control items (e.g., *soccer players*, for which the positive and negative adjective were endorsed equally as often $\beta = -0.34$ $[-1.04, 0.35]$). A comparable interaction was observed for categories that were expected to be near the low-end of the scale (e.g., jockey): The positive-form adjective was endorsed credibly less than the negative-form adjective, in comparison to the middle-of-the-scale subordinate categories: $\beta = -4.58$ $[-5.32, -3.84]$. Again, this was driven by the behavior of the low-end-of-the-scale subordinate categories, which showed strong endorsements for the negative than for the positive adjectives ($\beta = 4.92$ [4.22, 5.62]).

The results from the endorsement task were remarkably symmetric across the low-end vs. high-end of the scale subordinate categories. We observe for the control (middle-of-the-scale) subordinate categories (e.g., *soccer players*), a slight preference to reject both adjectives over accepting both (i.e., *neither tall nor short* was a more common pattern than

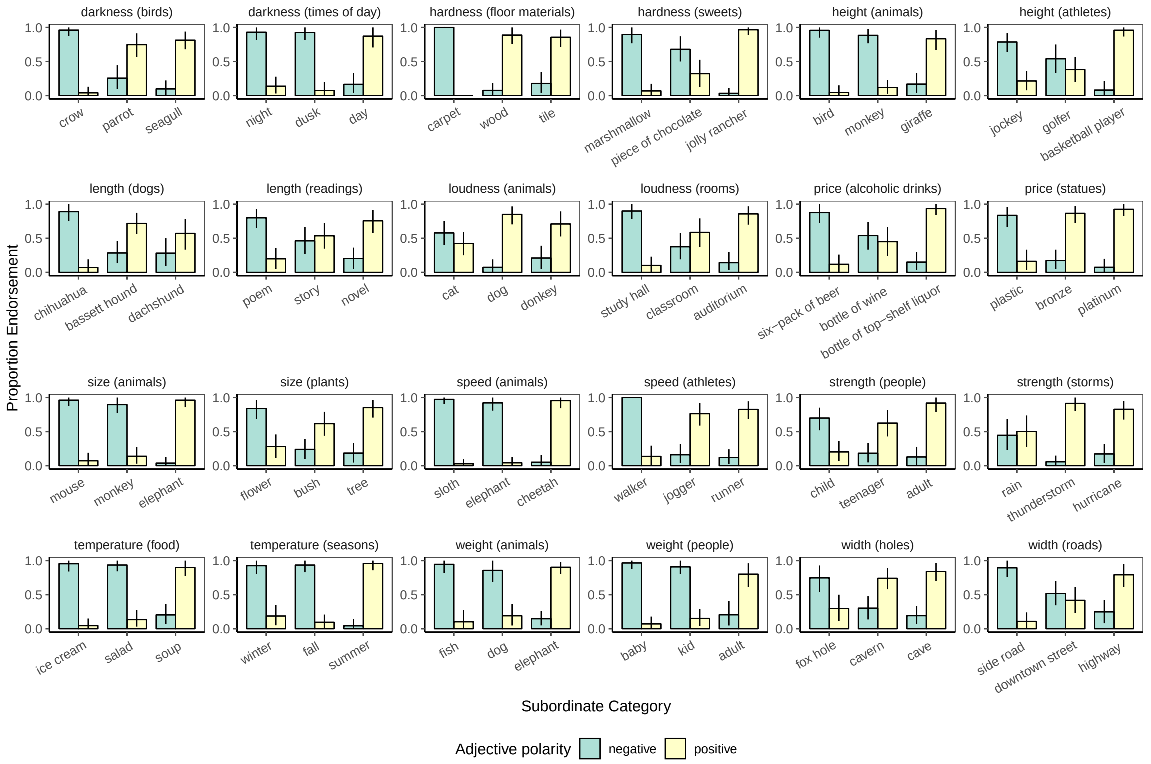

Figure S5. Task 3 (Adjective Endorsement) results for a subset of the items.

both tall and short; $\beta = -0.12$ $[-0.21, -0.02]$). The mean endorsements, averaging over positive and negative adjectives, for items at the high-end of the scale (e.g., *basketball players*) was not different from chance ($\beta = 0.01$ $[-0.09, 0.11]$) nor were the average endorsements of adjectives for categories at the low-end of the scale (e.g., *gymnasts*; $\beta = -0.02$ $[-0.11, 0.07]$). Furthermore, the endorsements of adjectives that are in conflict with a listener's general expectations of the categories (e.g., tall gymnasts vs. short basketball players) were not different between the low-end and high-end items ($\beta = 0.20$ $[-0.25, 0.65]$), nor were the inferences from adjectives that were consistent with general expectations about a category (e.g., short gymnasts vs. tall basketball players; $\beta = -0.26$ $[-0.71, 0.18]$).

Alternative Models

The inference about the comparison class outlined in the main text involves a listener reasoning about a speaker reasoning about a listener. The full Rational Speech Act model is given by these equations (reproduced from the main text):

$$L_1(x, c \mid u; k) \propto S_1(u \mid x, c) \cdot P(x \mid k) \cdot P(c \mid k) \tag{S1}$$

$$S_1(u \mid x, c) \propto \exp\left(\alpha \cdot (\ln L_0(x \mid u, c) - \text{cost}(u))\right) \tag{S2}$$

$$L_0(x \mid u, c) = \int_\theta L_0(x, \theta \mid u, c) \, \mathrm{d}\theta \tag{S3}$$

$$L_0(x, \theta \mid u, c) \propto \delta_{[\![u]\!](x,\theta)} \cdot P(x \mid c) \cdot P(\theta) \tag{S4}$$

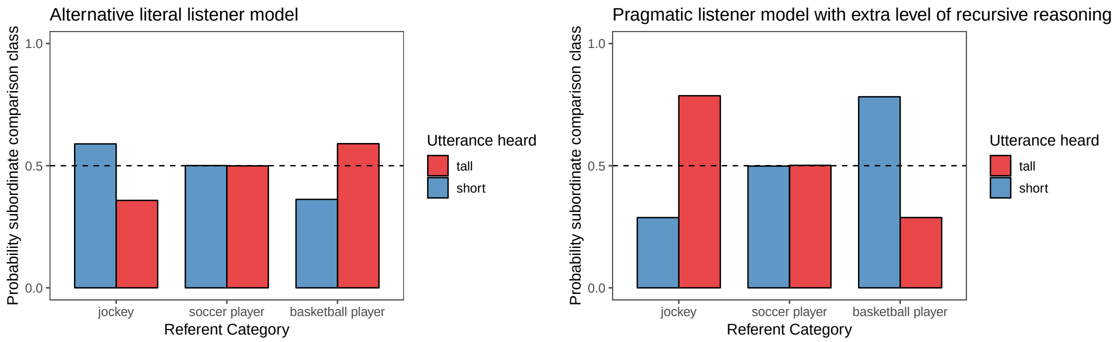

Figure S6. Alternative model predictions. A: A literal model that does not represent a speaker's representation of the context as separate from their own. This listener effectively answers the question of what is more likely: a basketball player who is tall or a person who is tall? This model predicts the exact opposite pattern of data from the pragmatic model reported in the main text. B: A more sophisticated pragmatic listener model than that reported in the main text. This model reasons about which comparison class a speaker believes that the listener would infer. This model produces the same pattern of results as the simpler pragmatic model reported in the main text.

Literal alternative model

One might question whether the inference about the comparison class is necessarily a pragmatic inference which requires recursive reasoning. We can investigate this question by reformulating the comparison class inference spelled out in Equation S1 in terms of a literal listener model (*a la* Equation S4). This literal comparison class inference model is given by:

$$L_0(x, \theta, c \mid u, k) \propto \delta_{[\![u]\!](x,\theta)} \cdot P(x \mid c) \cdot P(\theta) \cdot P(c \mid k) \quad \text{(S5)}$$

Similar to the pragmatic listener model (Eq. S1), this listener can use their knowledge of the referent k to constrain the hypothesis space of comparison classes (e.g., with the knowledge that the referent is a basketball player, consider comparison classes that the same as or superordinate to the class of basketball players). Unlike the pragmatic listener model (Eq. S1), however, the literal listener version of the model does not hold different representations of the referent in mind: The pragmatic listener has their private representation of the referent—given by the prior distribution of the degree $P(x \mid k)$—and imagines a speaker who acts assuming some comparison class—$S_1(u \mid c)$—where c and k may or may not index the same class (e.g., the listener may know the referent is a basketball player – k = *basketball players* – but believe the speaker was assuming a *person* comparison class – c = *people*). The literal listener version of the model has no way of separating these representations. In effect, this listener is answering a slightly different question from the comparison class inference problem. The question this alternative model is answering is: what is more likely—a basketball player whose height is greater than some threshold or a person whose height is greater than some threshold? This alternative model predicts the exact opposite pattern of results from the pragmatic listener model (Figure S6A).

Alternative pragmatic model

The pragmatic comparison class inference listener model (Eq. S1) reasons about which comparison class a speaker is more likely to be assuming. That is, the speaker (Eq. S2) is presumed to be assuming that a particular comparison class is already in the common ground, analogous to a presupposition (e.g., saying "My car is in the shop" presupposes that the speaker owns a car). Speakers may be aware, however, that the comparison class is not in the common ground; still, they may still avoid articulating a comparison class (e.g., uttering "He's short for a basketball player") if the listener can reasonably be assumed to infer the comparison class.

This kind of inference is more sophisticated: It involves a listener reasoning about the comparison class that a speaker believed the listener would infer. As demonstrated above with the Literal Alternative model, this first-order listener inference about the comparison class itself involves pragmatic reasoning. This higher-order pragmatic reasoning model is given by the following equations:

$$L_2(x, c \mid u, k) \propto S_2(u \mid x, c) \cdot P(x \mid k) \cdot P(c \mid k) \tag{S6}$$

$$S_2(u \mid x, c) \propto \exp\left(\alpha \cdot (\ln L_1(x, c \mid u) - \mathrm{cost}(u))\right) \tag{S7}$$

where L_1 is the pragmatic inference model defined above and in the main text (Eq. S1). The primary difference between this model and the model given by Eqs. S1 and S2 is in the speaker S_2 (Eq. S7) (vs. the S_1 shown in Eq. S2). S_2 chooses their utterance by taking into account the fact that the listener L_1 (Eq. S1) is uncertain about the comparison class. As shown in Figure S6B, this more sophisticated pragmatic inference model arrives at the same conclusions about the likely comparison class given different general expectations about the category and the adjective heard. Further, the inferences of this model, like those of the simpler pragmatics model, are resilient to reasonable choices of alternative utterances; most notably, if the set of alternative utterances provides a way to explicitly articulate the comparison class (e.g., the speaker could have said *They're tall for a basketball player*), the same inferences result from hearing the utterance without a comparison class.

Since this alternative pragmatic model derives the same qualitative inference for the contexts we study about the comparison class as the model presented in the main text, we do not compare the two models in the Quantitative Analysis section. It is likely that the predictions of these two models would come apart under different contextual manipulations of speaker and/or listener knowledge. For example, the model presented in the main text might be better suited for third-party conversations and its architecture could be useful for developing models of word learning from overheard speech (Akhtar, Jipson, & Callanan, 2001; Martínez-Sussmann, Akhtar, Diesendruck, & Markson, 2011). On the other hand, the more sophisticated pragmatic inference model presented in this supplement might be better suited for dyadic interactions with informed and intentional interlocutors.

Bayesian Data Analysis

We generate quantitative predictions for the Comparison Class Inference by constructing a joint Bayesian data-analytic model that synthesizes the data from the Comparison

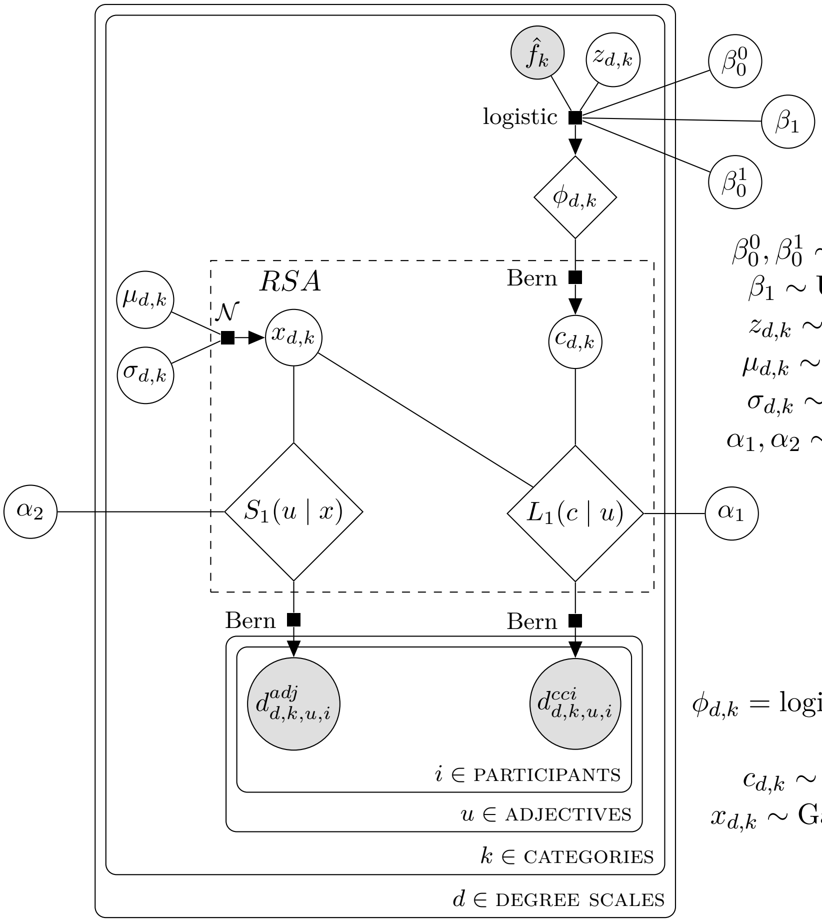

$$\beta_0^0, \beta_0^1 \sim \text{Gaussian}(0, 2)$$
$$\beta_1 \sim \text{Uniform}(-3, 3)$$
$$z_{d,k} \sim \text{Bernoulli}(0.5)$$
$$\mu_{d,k} \sim \text{Uniform(-3, 3)}$$
$$\sigma_{d,k} \sim \text{Uniform(0, 3)}$$
$$\alpha_1, \alpha_2 \sim \text{Uniform(0, 20)}$$

$$\phi_{d,k} = \text{logistic}(\begin{cases} \beta_0^0 + \beta_1^0 \cdot \hat{f}_k & \text{if } z_{k,d} = 0 \\ \beta_0^1 + \beta_1^1 \cdot \hat{f}_k & \text{if } z_{k,d} = 1 \end{cases})$$
$$c_{d,k} \sim \text{Bernoulli}(\phi_{d,k})$$
$$x_{d,k} \sim \text{Gaussian}(\mu_{d,k}, \sigma_{d,k})$$

Figure S7. Joint Bayesian data analytic strategy of the maximal model. Two related RSA models (the comparison class inference model L_1 and the adjective endorsement model S_1) directly predict the data from Tasks 2 & 3 (d^{cci} and d^{adj}), respectively. Each of these models relies upon world knowledge, which varies by the degree d (e.g., height) and category k (e.g., basketball players)—$P(x^{d,k})$, assumed to be Normal distributions with unknown mean μ_k^d and variance σ_k^d). The prior probability of a comparison class $c_{d,k}$ is used only in the L_1 model, and is assumed to be a logistic linear function encoding a basic level bias β_0 and an effect of frequency of the noun phrase $\hat{f}_k$ given by β_1. The referent category of each stimulus may be either a subordinate level category or a basic-level category, determined by parameter $z_{d,k}$, which gates between using two different basic-level bias comparison class parameters. Finally, each RSA model has its own speaker optimality parameter α.

Class Inference experiment (Task 2) and the Adjective Endorsement experiment (Task 3) using Rational Speech Act models to predict the data from each experiment. The RSA model for the comparison class inference data is described in the main text of the experiment and is given by Eq. S1.

We model the adjective endorsement data using the pragmatic speaker component S_1 (Eq. S2) of the comparison class inference model, with three slight modifications: (1) the comparison class c is assumed to be the superordinate comparison class, since the endorsement task concerns sentences drawing explicit comparisons to a superordinate class,

(2) the speaker has two alternative utterances: produce the adjective in question (e.g., tall or short) or stay silent (following Degen & Goodman, 2014; Tessler & Goodman, 2019), and (3) the speaker's utility is computed via an expectation about the degree value x for the referent given by its category membership k: $x \sim P_k$ (e.g., the speaker does not have a particular height in mind to convey but averages over their expectations about the height of the referent). The adjective endorsement RSA model we use is given by:

$$S_1(u \mid k) \propto \exp\left(\alpha_2 \cdot \mathbb{E}_{x \sim P_k} \ln L_0(x \mid u)\right) \tag{S8}$$

where $L_0(x \mid u)$ is Equation S4 printed above.

Each RSA model has a global speaker optimality parameter α_i which determines the degree to which speakers are assumed to be informative. This parameter is not of direct theoretical interest and we use priors consistent with the previous literature on RSA models: $\alpha_i \sim \text{Uniform}(0, 20)$.

Degree priors (World knowledge)

The two RSA models (Eq. S1 for the Comparison Class Inference task and Eq. S8 for the Adjective Endorsement task) depend upon the same world knowledge priors $P(x \mid k)$ (e.g., expectations about the height of basketball players), which vary as a function of the subordinate category k. Thus, we put uncertainty over these world knowledge prior distributions and infer the priors that best jointly explain the data from the two tasks.

Only the relative values for $P(x \mid c = c_{sub})$ and $P(x \mid c = c_{super})$ affect model predictions. We further assume the degree priors follow Gaussian distributions. Hence we fix each superordinate distribution to be a standard normal distribution $P(x \mid c = c_{super}) = \mathcal{N}(0, 1)$ and the subordinate priors are Gaussian distributions with unknown means and variances $P(x \mid c_{sub}) = \mathcal{N}(\mu_{sub}, \sigma_{sub})$; the subordinate priors thus have standardized units. We put priors over these mean and variance parameters of each subordinate category distribution k for each degree d: $\mu_k^d \sim \text{Uniform}(-3, 3)$, $\sigma_k^d \sim \text{Uniform}(0, 3)$, and infer their likely values using Bayesian Data Analysis (left-hand side of Figure S7).

Comparison class priors and description of alternative models

The comparison class prior $P(c)$ reflects listeners' expectations of what comparison classes are likely to be used in conversation. Baseline expectations about conceptual comparison classes could be a function of the level of abstraction of the categories in question as well as the usage frequency of the noun phrases used to describe those categories. For example, basic-level categories may be more probable conceptual comparison classes because of their utility in everyday reasoning (Rosch & Mervis, 1975); additionally, we might expect the relative probability of basic-level vs. subordinate level categories to differ from basic vs. superordinate categories. To investigate these possibilities, we construct and compare models which differ in how the comparison class prior is parameterized.

Flat prior model (baseline). Our baseline model makes inferences based only on distributional knowledge about scalar properties of categories $P(x \mid k)$ (described above). For this model, the comparison class prior is uninformed and does not provide an *a priori* preference for the relatively subordinate or relatively superordinate comparison class; thus,

all of the by-item variability in comparison class inferences in this model must be explained as a function of the distributional knowledge about properties.

Basic-level bias. The *basic-level bias* model assumes some *a priori* preference for the Subordinate or Superordinate comparison classes. We parameterize the comparison class prior via a logistic model, where a basic-level bias plays the role of an intercept term β_0: $P(c) = \text{logistic}(\beta_0)$. Our crowd-sourced stimuli generation procedure (Task 1) presents interesting inferential challenges for this analysis. *A priori*, we do not know if the NPs we use to introduce the referents (Referent NPs; those generated by participants in Task 1) are basic-level categories or subordinate level categories, and hence, whether the more general comparison class would correspond to a superordinate-level category or a basic-level category. A basic-level bias could plausibly operate differently for a subordinate vs. basic-level inference than for a basic vs. superordinate level inference. Specifically, superordinate comparison classes might be the most implausible, because superordinate categories are more heterogenous in comparison to basic-level or subordinate-level categories. Thus, we endow our data-analytic model with two regression coefficient parameters corresponding to the intercept term (i.e., the basic-level bias term), and introduce a Bernoulli random variable z for each NP to indicate whether it is a subordinate-level term or basic-level term (Figure S7).

Frequency effect. The *frequency effect* model assumes that the NPs that denote comparison classes which are more common in usage could have a higher *a priori* probability of serving as the comparison class. The comparison class prior in this model is thus a (logistic) function of the relative usage frequency f_k of the subordinate NP vs. superordinate NP estimated from the Google WebGram corpus[2]: $P(c) = \text{logistic}(\beta_1 \cdot \log(\frac{\hat{f}_{sub}}{\hat{f}_{super}}))$. We assume a usage-based frequency effect to operate independent of the level-of-abstractness of the category; hence, we do not infer separate β_1's for the imputed subordinate vs. basic-level categories.

Full model: Basic-level bias and frequency effect. To test whether both a basic-level bias and frequency effect jointly contribute to the comparison class prior, our full model assumes both contribute via a logistic-linear model: $P(c) = \text{logistic}(\beta_0 + \beta_1 \cdot \log(\frac{\hat{f}_{sub}}{\hat{f}_{super}}))$.

Priors for comparison class parameters. The priors over the regression parameters (which also apply to the alternative models described above, if they include that parameter) are $\beta_0^0, \beta_0^1 \sim \text{Gaussian}(0, 2)$, $\beta_1 \sim \text{Uniform}(-3, 3)$. The binary level-of-abstractness parameter which governs the direction and magnitude of the basic-level bias comes from a uninformed prior: $z_k \sim \text{Bernoulli}(0.5)$.

Model implementation

We ran four different BDA models, corresponding to the different ways of parameterizing the comparison class prior: (1) Flat Prior model (assumes the comparison class prior is always 50/50 between subordinate and superordinate class); (2) Basic-level bias, or intercept only model (assumes a basic-level bias), (3) Frequency effect, or slope only model (assumes an effect of corpus frequency, but no basic-level bias), (4) Basic-level bias

[2] Corpus accessed via `https://corpora.linguistik.uni-erlangen.de/cgi-bin/demos/Web1T5/Web1T5_freq.perl`

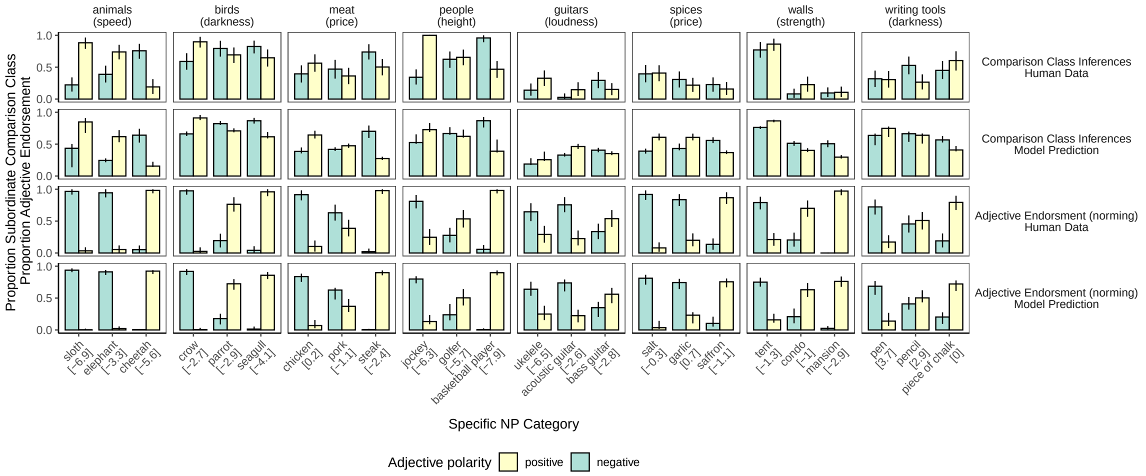

(a) Human data and model predictions for items with small residuals (left 4) and largest residuals (right 4) for comparison class inference data. Top two rows show data and model predictions for the main Comparison Class Inference experiment; bottom two rows correspond to the Adjective Endorsements (norming study). The log ratio frequency of subordinate NP to superordinate NP is shown in brackets next to the x-axis labels; a more negative number corresponds to a stronger prior belief in the superordinate category as the comparison class.

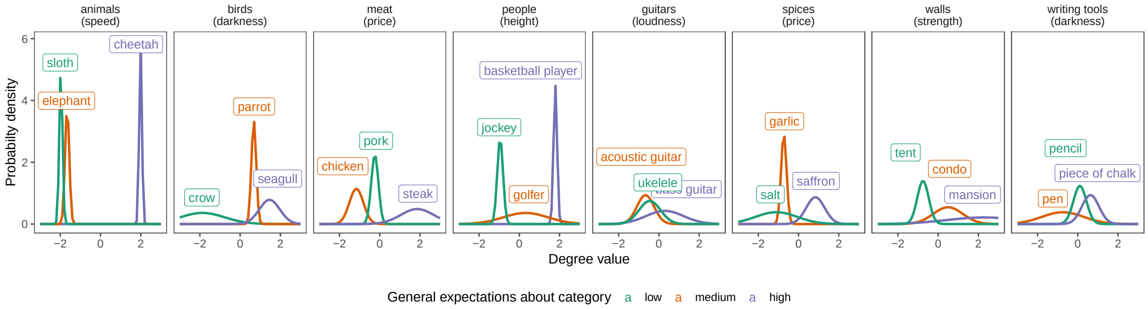

(b) Imputed prior distributions over degrees for 8 of the 90 item sets. These distributions were generated from the Maximum A-Posteriori parameter values inferred by conditioning on the Comparison Class Inference and Adjective Endorsement data sets.

Figure S8. Quantitative modeling results for eight sets of items which show the lowest and highest residuals for the comparison class inference data.

and Frequency effect (slope and intercept). Figure S7 shows the plate diagram corresponding the BDA model for the full, slope and intercept model. We implemented the RSA and Bayesian data analysis models in the probabilistic programming language WebPPL (Goodman & Stuhlmüller, 2014) and performed inference by running 7 MCMC chains with 500,000 iterations each, discarding the first 250,000 for burn-in. Convergence was checked through visual inspection of the different chains to ensure similar conclusions would be drawn from each chain independently.

We estimated the marginal likelihood of the data under each model by using an Annealed Importance Sampling algorithm implemented in WebPPL (Neal, 2001). We collected 4 samples from the AIS algorithm, which was run 250,000 steps per sample.

Supplementary model results

Figure S8 shows model predictions and inferred parameters for four sets of items which for which the full model does well at predicting the comparison class inference data and four sets of items where the full model does not do well at predicting the comparison class inferences. Predictions in the Comparison Class Inference task (Fig. 8(a), top rows) can be triangulated by examining the predictions in the Adjective Endorsement task (Fig. 8(a), bottom rows) and the inferred world knowledge parameters (Fig. 8(b)).

References

Akhtar, N., Jipson, J., & Callanan, M. A. (2001). Learning words through overhearing. *Child development*, *72*(2), 416–430.

Degen, J., & Goodman, N. (2014). Lost your marbles? the puzzle of dependent measures in experimental pragmatics. In *Proceedings of the annual meeting of the cognitive science society* (Vol. 36).

Goodman, N. D., & Stuhlmüller, A. (2014). *The Design and Implementation of Probabilistic Programming Languages.* `http://dippl.org`. (Accessed: 2019-9-19)

Martínez-Sussmann, C., Akhtar, N., Diesendruck, G., & Markson, L. (2011). Orienting to third-party conversations. *Journal of Child Language*, *38*(2), 273.

Neal, R. M. (2001). Annealed importance sampling. *Statistics and computing*, *11*(2), 125–139.

Rosch, E., & Mervis, C. B. (1975). Family resemblances: Studies in the internal structure of categories. *Cognitive psychology*, *7*(4), 573–605.

Tessler, M. H., & Goodman, N. D. (2019). The language of generalization. *Psychological review*, *126*(3), 395.
